# A magnetically recoverable, fluorescence-tracked, molecularly imprinted nanocarrier for controlled bortezomib release

**DOI:** 10.1186/s11671-026-05039-x

**Published:** 2026-11-12

**Authors:** Taha Majid Mahmood Sheikh, Zeeshan Ali, Hafiz Muhmmad Usman Abid, Muhammad Hayat, Mehwish Raza

**Affiliations:** 1 School of Medical Sciences, Shandong Xiehe University, Jinan, 250109 P. R. China; 2 Department of Chemistry NFC Institute of Engineering & Technology, Multan, 60000 Pakistan; 3 Faculty of Pharmacy, Bahauddin Zakariya University, Multan, 60800 Pakistan; 4 Institute of Chemical Sciences, Bahauddin Zakariya University, Multan, 60800 Pakistan; 5 Multidisciplinary Research Lab, Bahria University Health Sciences Campus Karachi, Karachi, Pakistan

**Keywords:** Stimuli-responsive drug delivery, Dual fluorescent nanoparticles, Anticancer drugs, Bortezomib, Molecularly imprinted polymers

## Abstract

**Background:**

A dual fluorescent-magnetic molecularly imprinted nanocomposite (FMMIP-BTZ) was synthesized by coating Fe₃O₄ nanoparticles with silica and fluorescein-5-isothiocyanate, followed by in-situ polydopamine polymerization using bortezomib (BTZ) as template.

**Methods:**

The prepared nanocomposite was characterized using scanning electron microscopy (SEM), transmission electron microscopy (TEM), thermogravimetric analysis (TGA), vibrating sample magnetometry (VSM), X-ray diffraction (XRD), fourier transform infrared (FTIR), UV-visible and fluorescence analysis.

**Results:**

Template-selective recognition was confirmed by an imprinting factor of 3.20 (85.34 mg g⁻¹ for FMMIP versus 26.67 mg g⁻¹ for the non-imprinted control), with a BTZ loading efficiency of 69.3%. Release followed zero-order kinetics (k₀ = 3.276% h⁻¹, R² = 0.9937), reaching 80.5 ± 1% cumulative release, and was pH-responsive, rising from 41.1% at pH 7.4 to 84.5% at pH 4 over 6 h. Saturation magnetization fell from 0.48 to 0.04 emu g⁻¹ as the non-magnetic shell was added; despite this, particles remained separable under an external magnetic field at every synthesis, washing, and release step. Dual fluorescence from released BTZ and the FITC-labelled core enabled spectroscopic tracking of release. In an acute dermal irritation assessment, FMMIP-BTZ produced a mean erythema score of 1.00 at 24 h compared with 3.00 for the control. Molecular docking against COX-2, PRDX5, EGFR, and AKT1, together with a 100-ns MD simulation of the AKT1–bortezomib complex, provided exploratory mechanistic context for the experimentally observed response.

**Conclusion:**

FMMIP-BTZ is thus a molecularly selective, magnetically separable, fluorescence-trackable platform with controlled, pH-responsive release; cellular uptake, cytotoxicity, and in vivo antitumour efficacy remain to be established.

**Supplementary Information:**

The online version contains supplementary material available at https://doi.org/10.1186/s11671-026-05039-x.

## Introduction

The global cancer burden remains substantial, with approximately 20 million new cancer cases and 9.7 million cancer-related deaths estimated worldwide in 2022 [1]. Earlier projections suggest that the number of new cancer cases could reach approximately 28.4 million annually by 2040 [2]. Therefore, much attention is being devoted to targeted delivery and controlled release of drugs. Such drug delivery systems (DDSs) should have the significant potential to lower the required therapeutic dose through more targeted therapy [3, 4]. Among many of the drug delivery systems, nanoparticles-based DDS are frequently used due to their several attributes such as specificity of targeted delivery, appropriate size (approx 3 – 200 nm) for extended circulation time in the vessels, increasing the drug concentration in intracellular medium of cancer cells, and lowering the toxic effects in healthy cells [5, 6]. The nanoparticles used in DDSs are usually synthesized from dendrimers [7], viruses [8], functionalized carbon nanotubes [9], polymeric nanoparticles/nanocomposites [10], micelles [11], and liposomes [12]. Among these materials, polymers have contributed considerably to the development of DDSs because they are customizable to deliver and control the release of drugs in various ways [13].

Polymeric drug delivery systems are categorized by their activation into groups, including chemical activation (biodegradation), solvent activation, diffusion control, and temperature-/pH-sensitive systems [14, 15]. A class of polymeric DDSs known as molecularly imprinted polymers (MIPs) can be developed using several polymerization techniques, including emulsion [16], precipitation polymerization [17], and dispersion [18]. The performance of MIPs is strongly influenced by polymerization conditions, including temperature, solvent properties, polymerization-mixture volume, initiation conditions, and polymerization time [19]. When the templates are removed from the polymeric matrix after cross-linkers are formed around the monomer-template complexes, certain cavities are created based on the templates’ size, shape, and functional group [20, 21]. The essential factors in constructing MIPs are the selection of the most suitable template, the tuning of strength, and the nature of the cross-linker [22]. An appropriate template (such as bortezomib, an anti-cancer medicine) can form a complex with monomers during polymerization that can be applied for drug delivery. Indeed, there have been some good achievements in the selection of specific anti-cancer medicines such as 5-fluorouracil (5-FU), docetaxel (DTX), doxorubicin (DOX), cisplatin (CP), and bortezomib (BTZ) for targeted and controlled drug release systems [23, 24].

The development of a novel drug delivery system with particular advantages can be facilitated by innovative technology and functional carriers and/or materials. Nanocarriers (modified or unmodified) can help reduce non-specific adsorption while allowing extension of the half-life of drugs in blood serum [25]. In this respect, an effective DDS must include components resistant to erosive forces and offer enteric solubility and hydrophobicity [26]. However, only a few DDSs have received clinical approval based on their therapeutic effectiveness in delivering medicines with minimal side effects [27].

A 5-FU-coated magnetic imprinted polymer was introduced to treat mice with liver cancer in 2016 [28]. In a similar study, a docetaxel-coated fluorescent magnetic MIP was also synthesized and applied to treat liver cancer in mice [24]. Although these studies support the validity of MIPS in DDSs, the most widely used chemicals/precursors for MIP synthesis suffer limitations. For instance, several chemicals frequently used in combination polymerization may pose certain health hazards in their handling (e.g., acrylic acids, azobisisobutyronitrile, methacrolyl chloride, and toluene) [29, 30]. Mild exposure to acrylic acid may cause skin, eyes, and mucous membrane irritation in humans, whereas azobisisobutyronitrile can be explosive upon decomposition above 65 °C [31]. Accordingly, it is a priority to secure appropriate monomers and crosslinkers to synthesize green and eco-friendly MIPs [32]. Recent work has continued to expand the scope of molecularly imprinted and magnetic nanomaterial platforms for selective extraction and delivery applications. A thermosensitive molecularly imprinted polymer was recently developed for the selective extraction of the anticancer drug imatinib mesylate from biological fluids, demonstrating the continued relevance of MIP-based selective recognition for oncology-related small molecules [33]. Another thermosensitive polymer brushes grafted onto magnetic nano-particles for extraction and determination of venlafaxine in biological and pharmaceutical samples is also investigated [34]. Similarly photoresponsive molecularly imprinted polymer on magnetic nanoparticles is also used for determination of methylprednisoloneacetate in pharmaceutical samples [35]. Dopamine is recognized as a green monomer and can self-polymerize in an alkaline environment [36]. During polydopamine (PDA) formation, different functional groups (i.e., amine and hydroxyl) become part of the material’s structure, making it able to attract water which in turn allow the material to form bonds with water and interact with it. This creates a suitable environment for the target substances to move through the drug carrier, making it easier for them to reach the specially-made spaces that are meant to hold them. This improved access is crucial for the effective binding and recognition of the target substances [37, 38]. Additionally, polydopamine-based MIPs can be synthesized for DDS applications with good biodegradability and biocompatibility [28]. Dopamine coated MIP is often extremely thin, less than 10 nm thick, which makes it well-suited for precise drug delivery and molecular detection. Furthermore, the ability of dopamine coated MIP to adjust its shape and thickness offers promising advancements in drug delivery systems and molecular sensing technologies. As such, incorporating fluorescent material into nanocarriers is expected to play a pivotal role in DDS applications for in vitro and in vivo anticancer treatment [27].

**Fig. 1:**
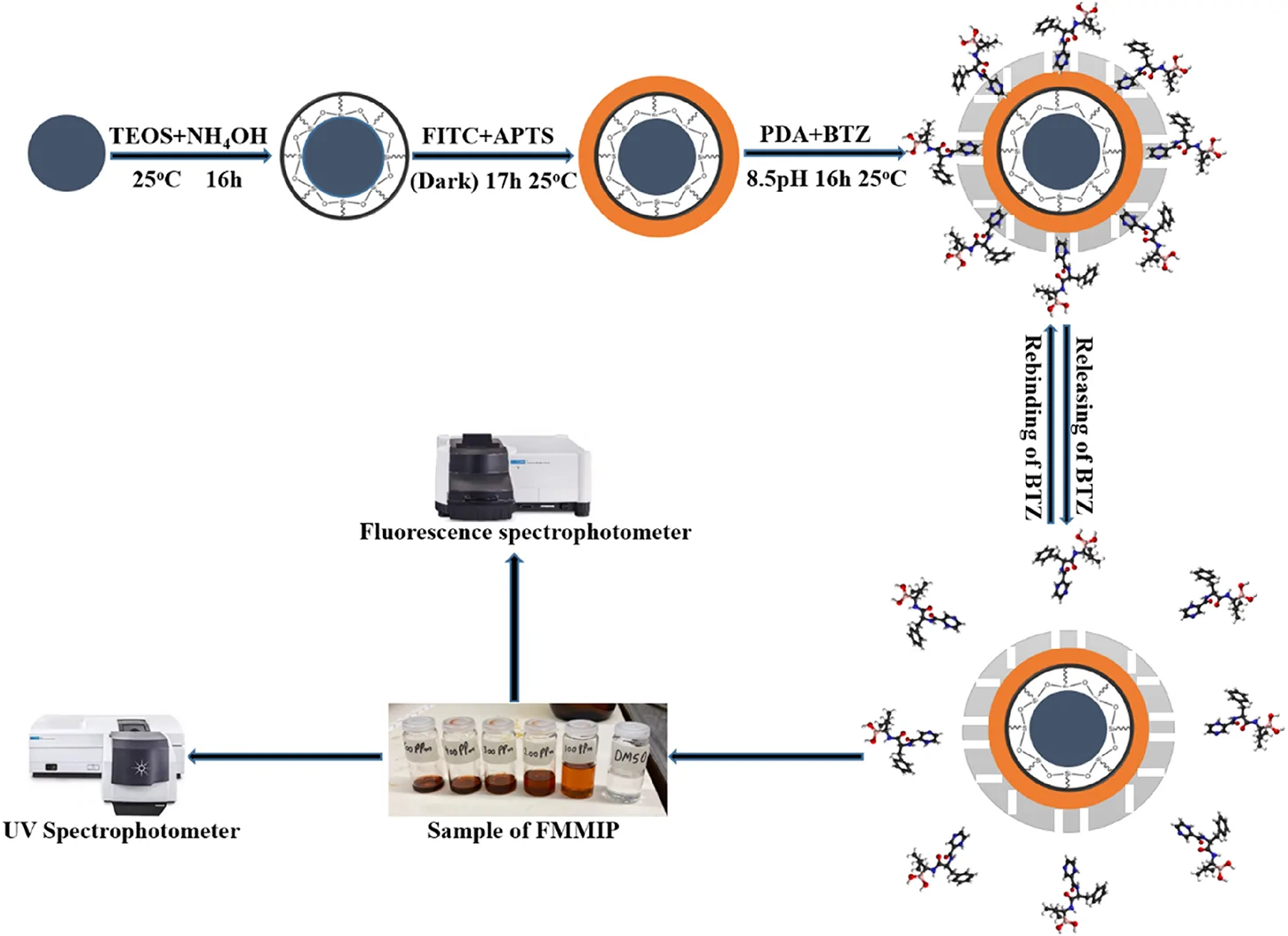
Schematic illustration of the synthesis of FMMIP-BTZ and BTZ releasing from FMMIP

The present study combines multiple functional components on the same nanoplatform by coating the magnetic core of Fe₃O₄ with a polydopamine layer functionalized with bortezomib, a molecule of interest, and fluorescence functionality based on FITC. The functional components of the molecularly imprinted drug delivery system (MIDDS) are multiple; in the present study combining a bortezomib imprinted polydopamine coating, an Fe₃O₄ magnetic core and a fluorescence functionality based on FITC. This multifunctional design allows preferential molecular recognition of BTZ, monitoring of drug release using fluorescence, magnetic recovery, and pH-dependent controlled release behavior. In addition, comparative evaluation of molecularly imprinted and non-imprinted polymers, preliminary dermal compatibility evaluation and computational investigations, including molecular docking and molecular dynamics (MD) simulations, have been carried out. The novelty of this work is not only in the development of a traditional drug delivery system, but also in the development of an integrated molecule-imprinted nanocarrier that acts as a fluorescent tag and a magnetic particle. The schematic of the synthesis of FMMIP-BTZ and its releasing and primary dermal irritation index studies are shown in Fig. 1.

## Materials and methods

### Reagents and materials

Chemicals used in this work were obtained from Sigma Aldrich and Merck as analytical grade: iron (III) chloride hexahydrate (FeCl_3_.6H_2_O), iron (II) sulfate (FeSO_4_), tri-sodium citrate (Na_3_C_6_H_5_O_7_), tetraethyl orthosilicate (TEOS), ammonia solution, acetone, ethanol, fluorescein-5-isothiocyanate (FITC), 3-aminopropyltriethoxysilane (APTS), dopamine hydrochloride, 2-amino-2-(hydroxymethyl)1,3 propanediol, hydrochloric acid (HCl), sodium chloride (NaCl), potassium chloride (KCl), disodium hydrogen phosphate (Na_2_HPO_4_), potassium dihydrogen phosphate (KH_2_PO_4_), and bortezomib. They were used without further purification.

### Animal source and treatment

The albino rats of the Primary Dermal Irritation Index (PDII) study were housed in the Pharmacy Department animal house, Bahauddin Zakariya University, Multan, Pakistan. Animals were between 10 and 12 weeks old, weighing 300–350 g. Before the experimental procedure, the animals were anaesthetized using ketamine (80 mg kg^−^¹) and xylazine (8 mg kg^−^¹) injected intraperitoneally. Induction of anaesthesia was achieved about 10–15 min prior to the procedure. The pedal withdrawal reflex was not observed before the experimental manipulation, indicating adequate anesthesia. During the experimental period, no changes were observed in the animals, and the PDII response was assessed during 24 h. Following completion of the 24 h observation period, the animals were allowed to recover from anesthesia and were returned to the animal facility. All procedures were carried out following the protocol approved by the Pharmacy Ethical Committee, Faculty of Pharmacy, Bahauddin Zakariya University, Multan, Pakistan and American Veterinary Medical Association.

### Synthesis of iron nanoparticles (Fe₃O₄ NPs)

Fe₃O₄ nanoparticles were prepared by co-precipitation of iron (II) and iron (III) salts (molar ratio 1:2) in the presence of 25% ammonia solution following a reported procedure [39]. Briefly, a solution of 10 mmol of iron sulfate and 20 mmol of iron chloride in 140 mL of distilled water was mixed in a two-necked round bottom flask under N_2_ at 70 °C for 30 min. Then 33 mL of ammonia solution (25%) was added dropwise until the formation of iron oxide NPs. Afterwards, the obtained product was separated using an external magnetic field and washed several times with distilled water and ethanol to a neutral pH. The precipitate was dried under vacuum at room temperature overnight. The separated NPs were redispersed in 200 mL deionized water with 0.3 M trisodium citrate and heated at 70 °C for 2 h. The NPs were washed with acetone to remove the excess citrate and dried in a desiccator for 15 h.

### Coating of silica core on iron nanoparticles (Fe₃O₄ @SiO₂ NPs)

The Fe₃O₄ NPs were further modified with silica to gain stability while limiting aggregation. Fe₃O₄ @SiO₂ NPs were prepared according to Stober’s method [40]. Fe₃O₄ NPs (solution A) were dispersed in 50 mL of deionized water in a sonication tub. An aliquot of 2 mL of water-dispersed NPs was diluted with 40 mL of deionized water. Then, a mixture of ammonia solution 25% (5 mL) and ethanol (140 mL) was added and stirred mechanically for 18 h. A solution (B) of 20 mL of ethanol and 1 mL of TEOS was added dropwise to diluted ferrofluid solution A, and the reaction was allowed to continue for 16 h at room temperature. Finally, the brownish-black product was separated with an external magnetic field, washed three times with ethanol and deionized water, and dried in a vacuum desiccator.

### Covering of fluorescent layer on silica core shell

The FITC-coated luminescent nanoparticles, Fe₃O₄@SiO₂@FITC (core-shell), were synthesized using a published method [41, 42]. Firstly, a solution of FITC (0.04 g) in 10 mL ethanol with APTS (0.22 mL) was stirred for 24 h in the dark while wrapped in aluminum foil. Then, the FITC solution was mixed with Fe₃O₄@SiO₂ NPs and stirred mechanically for 17 h to obtain Fe₃O₄@SiO₂@FITC (core-shell), which were dried at room temperature in a vacuum and stored in amber glass vials.

### Synthesis of MIPs and NIPs

The imprinted PDA layer was prepared by alkaline oxidative self-polymerization of dopamine in Tris buffer [43, 44]. Briefly, 80 mg of fluorescent Fe₃O₄@SiO₂@FITC core-shell, 80 mg of dopamine hydrochloride, and 15 mg of bortezomib were added into 40 mg of tris buffer (2-amino-2-hydroxymethyl 1,3 propanediol) solution to dissolve in 40 mL of deionized water, and the pH was adjusted to 8.5 by HCl (1 M). The above reaction was allowed to proceed at room temperature for 16 h with stirring at 200 rpm. Finally, the resulting magnetic material was obtained as MIP from the reaction of FITC-coated silica iron oxide nanoparticles and dopamine as a monomer for polymerization on the surface of Fe₃O₄@SiO₂@FITC core-shell and bortezomib as a template. The incorporation of BTZ on the polydopamine layer during synthesis was due to a combination of non-covalent interactions (π–π stacking, boronate-ester-type interaction and hydrogen bonding). The synthesized fluorescent-molecularly imprinted polymer (FMMIP-BTZ; Fe₃O₄@SiO₂@FITC@PDA-BTZ) was separated under an external magnetic field (EMF) and washed with deionized water and ethanol three times to remove the non-bound BTZ. The same procedure was applied to synthesize non-imprinted polymer but in the absence of a bortezomib template [44, 45].

The unbound and loosely adsorbed BTZ on the surface of FMMIP was removed through magnetic separation in an external magnetic field. After that, FMMIP was washed three times with ethanol and deionized water, leaving surface exposed recognition cavities complementary to BTZ. The efficiencies of this process were indirectly verified by calculating the imprinting factor (IF) ratio of the equilibrium adsorption capacities of FMMIP and the corresponding non-imprinted polymer (FMNIP) for BTZ. The equilibrium adsorption capacity of FMMIP was 85.34 mg/g and the adsorption capacity of FMNIP was 26.67 mg/g, which was 3.20 times that of FMMIP (Sect.  3.13, α = 3.20). An imprinting factor greater than 1 provides evidence of successful molecular imprinting and the availability of template-selective recognition sites after the washing process [22].

### 6% release of bortezomib from FMMIP and pH-dependent release study

Ex-vivo permeation studies were conducted for FMMIP-BTZ (*n* = 3) using a Franz Diffusion cell, and the average values were recorded. The experiment involved placing fresh rat skin between the donor and receptor chambers of the vertical cell setup. Before the study, the receptor chamber was filled with 5 mL PBS (pH 7.4), maintained at 37 °C, and continuously stirred at 100 rpm for 24 h. The formulation was then applied to the donor chamber. At specified time points, 1 mL aliquot samples were withdrawn from the medium, and an equal volume of fresh medium was replenished. The collected samples were subsequently diluted and analyzed using a spectrophotometer.

To investigate the pH-responsive controlled drug release behavior of Fe₃O₄@SiO₂@FITC@PDA-BTZ, 25 mg of FMMIP – BTZ was dissolved in each of three buffer solutions with pH values of 4, 5.5, and 7.4. Afterwards, the soluble drug BTZ was separated carefully under an EMF. The remaining MMIP NPs without BTZ were washed with ethanol and placed in a vacuum desiccator for two days. The MMIP NPs were weighted again, and each pH’s weight loss was calculated.

### Primary dermal irritation index

In albino rats, the Primary Dermal Irritation Index (PDII) was assessed using five groups, each consisting of four rats (*n* = 4 animals per group). The control group had a 2 cm² area of the abdominal skin treated with an ammonia solution. The second group was treated with 1 mL of 50 ppm Fe₃O₄, while the third group received 1 mL of a core-shell formulation. The fourth group’s skin was exposed to 1 mL of 50 ppm of the test drug (BTZ), and the fifth group was administered with 1 mL of 50 ppm FMMIP-BTZ. Erythema was observed at intervals 0, 8, and 24 h. Skin sensitization was evaluated, repeated three times, and the average values and standard deviations were recorded. The severity of erythema was categorized based on mean scores: 1 indicated no erythema, 2 represented light pink, 3 indicated dark pink, and 4 denoted dark red changes in the skin.

### Molecular docking studies

Molecular docking studies were conducted to provide exploratory mechanistic context for experimental results of in-vivo assessment of erythema. The molecular docking studies were performed using AutoDock Vina [46]. All the ligands including nanocomposites were sketched and optimized using the programs MarvinSketch [47] and ChemSketch [48]. The structures of fluorescein-5-isothiocyanate (FITC) having PubChem ID = 18,730, tetraethyl orthosilicate (TEOS) with PubChem ID = 6517, bortezomib (PubChem ID = 387447), and dopamine (PubChem ID = 681) were retrieved from PubChem database (https://pubchem.ncbi.nlm.nih.gov). The ligands were also categorized into the same groups as were during the in-vivo studies i.e., ammonia, Fe₃O₄, bortezomib and FMMIP-BTZ. Four different proteins involved in inflammation and skin irritation were selected as drug targets including cyclooxegenase-2 (COX-2), peroxiredoxin-5 (PRDX-5), epidermal growth factor receptor (EGFR) and AKT1. The three dimensional structures were retrieved from RCSB protein data bank having PDB IDs 5F1A, 1HD2, 5Y9T, and 4EJN respectively. All the protein structures and ligands were optimized using the AutoDock tools [49]. All the ligands were docked into the active site of each protein successively. The results of molecular docking studies were analyzed using Biovia Discovery Studio Visualizer 2016 [50]. The results of the molecular docking studies were expressed as binding affinity in kcal/mol. The lesser the binding affinity greater is the interaction of a ligand with the respective drug target.

### Molecular dynamics simulation

To validate and extend the molecular docking findings at the dynamic level, molecular dynamics (MD) simulations were performed using OpenMM 8.0 [51]. Based on the docking results, FMMIP-BTZ exhibited the highest binding affinity (− 11.0 kcal/mol) against AKT1 (serine/threonine kinase; PDB ID: 4EJN) among all four protein targets evaluated. Accordingly, AKT1 was selected as the simulation target. Since FMMIP-BTZ is a multicomponent nanocomposite and its complete three-dimensional structure cannot be retrieved as a single entry from a structural database, the simulation was performed using bortezomib (BTZ; PubChem CID: 387447) as the representative ligand, as it is the pharmacologically active template molecule whose structure is fully defined and publicly available on PubChem [52]. The crystal structure of AKT1 in complex with AKT inhibitor 12j (PDB ID: 4EJN) was retrieved from the RCSB Protein Data Bank (https://www.rcsb.org). All crystallographic water molecules, co-crystallized ligands, and heteroatom groups were removed prior to simulation preparation; only structurally relevant cofactors were retained. The protein was parameterized using the AMBER14-SB force field, and ligand (bortezomib) parameters were assigned using the Generalized Amber Force Field 2 (GAFF2) with AM1-BCC partial charges computed through the OpenFF toolkit.

The AKT1–bortezomib complex was solvated in an explicit TIP3P water box with a minimum padding of 10 Å on all sides [53]. Sodium (Na⁺) and chloride (Cl⁻) counter-ions were introduced to achieve charge neutrality and to replicate physiological ionic strength at 0.15 M. Energy minimization was carried out using the L-BFGS algorithm until the maximum force on any atom was reduced below 10 kJ/mol/nm. The minimized system was equilibrated in two consecutive stages: (i) NVT ensemble at 310 K for 500 ps with harmonic positional restraints (force constant = 1000 kJ/mol/nm²) on all protein and ligand heavy atoms, followed by (ii) NPT ensemble at 310 K and 1 atm for 1 ns with restraints progressively released. Temperature was maintained using a Langevin thermostat (collision frequency = 1 ps⁻¹), and pressure was regulated by a Monte Carlo barostat (pressure coupling interval = 25 steps). Following equilibration, a production MD simulation of 100 ns was conducted in the NPT ensemble using a 2-fs time step [54]. Covalent bonds involving hydrogen atoms were constrained with the LINCS algorithm. Long-range electrostatics were computed using the Particle Mesh Ewald (PME) method with a real-space cutoff of 10 Å, and the same cutoff was applied for Lennard-Jones van der Waals interactions. Trajectory frames were saved every 10 ps, yielding 10,000 frames for analysis. Post-simulation analyses were performed using MDTraj and MDAnalysis in Python, and included: backbone Cα root mean square deviation (RMSD), per-residue root mean square fluctuation (RMSF), radius of gyration (Rg), protein–ligand hydrogen bond counting (donor–acceptor distance ≤ 3.5 Å; angle ≥ 150°), and solvent-accessible surface area (SASA).

### Characterization

A Perkin-Elmer Nicolet iS5 FT-IR (Thermo Scientific, USA) instrument was used to record attenuated total reflection (ATR) infrared spectra in the range of 4000–500 cm⁻¹ with a resolution of 4 cm⁻¹ and 16 scans at room temperature. A Quanta Master spectrofluorometer C-60/2000 (Photon Technology International, Lawrenceville, NJ, USA) was used to detect fluorescence emissions. A Cary 60 UV/vis spectrophotometer (Agilent Technologies, USA) was used to record UV-vis absorption spectra. A scanning electron microscope (SEM LEO 1560, Thermal Field Emission Zeiss, Oberkochen, Germany) equipped with EDX (Mikroanalytisches Laboratorium Kolbe, Mülheim an der Ruhr, Germany) and a transmission electron microscope (JEOL 3000 F, Tecnai Spirit BioTWIN, FEI Company, OR, USA) were used to examine the surface morphologies of Fe₃O₄ nanoparticles, silica-coated iron nanoparticles (core-shell), and FMMIP-BTZ. For investigating the thermal properties, a thermal gravimetric analysis was performed by heating to 900 °C at a heating rate of 10 °C per min (TGA Q5000 V3.17 Build 265: Oberkochen, Germany). An elemental analyzer 1108 (Carlo-Erba, Milan, Italy) was used to analyze C, H, and N contents. X-ray diffraction analysis was performed (powder diffractometer Brucker D8, Germany) with CuKα radiation at 40 kV and 40 mA for 2θ = 20 – 80˚.

### Imprinting factor

The imprinting factor (α) for BTZ was determined by comparing the equilibrium adsorption capacities of the fluorescent magnetic molecularly imprinted polymer (FMMIP) and the corresponding fluorescent magnetic non-imprinted polymer (FMNIP). The imprinting factor was calculated using Eq. (1):

1 $$\alpha={\rm{q}_{m}}/{\rm{q}_{n}}$$

where qₘ is the equilibrium adsorption capacity of BTZ on the fluorescent magnetic molecularly imprinted polymer (FMMIP) (mg g⁻¹), and qₙ is the equilibrium adsorption capacity of BTZ on the corresponding fluorescent magnetic non-imprinted polymer (FMNIP) (mg g⁻¹). Both qₘ and qₙ were determined under identical equilibrium conditions.

### Statistical methods statement

Percentage release of bortezomib and primary dermal irritation index were performed in triplicate, and results are reported as mean ± standard deviation. Comparison of the binding capacities of FMMIP vs. FMNIP was (*n* = 5). No formal inferential statistical tests (e.g., ANOVA) were performed to compare groups in this study; comparisons are descriptive.

## Results and discussion

### FT-IR analysis

**Fig. 2:**
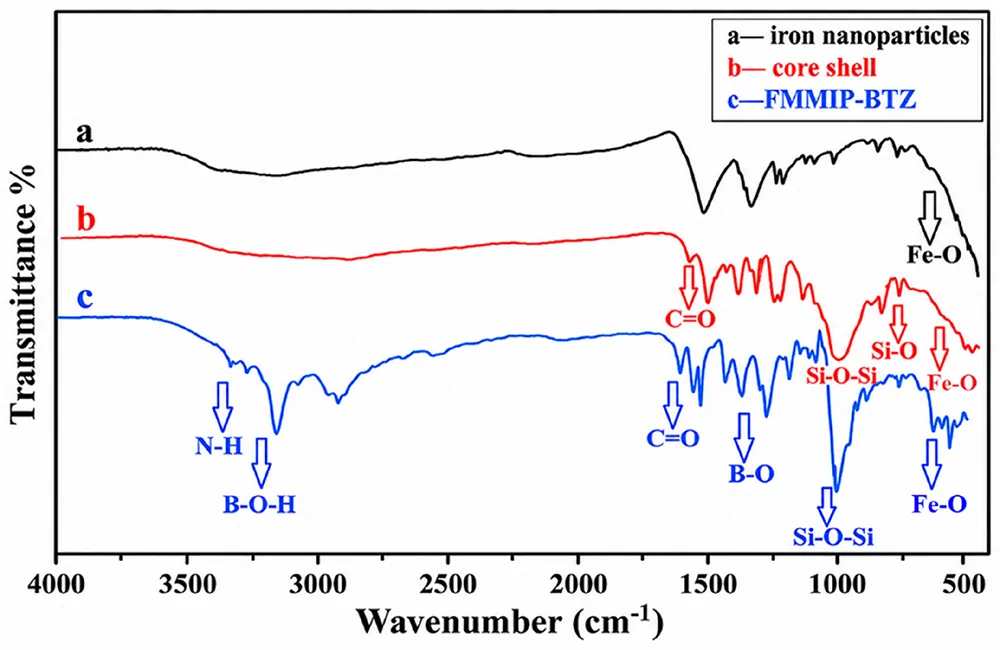
FTIR spectra of synthesized materials: (**a**) Fe₃O₄, (**b**) core-shell, and (**c**) FMMIP-BTZ

The FT-IR spectra of Fe₃O₄ NPs, core-shell Fe₃O₄@SiO₂@FITC core-shell, and FMMIP-BTZ nanocomposite (Fe₃O₄@SiO₂@FITC@PDA-BTZ) are presented in Fig. 2a and b, and 2c, respectively. The spectra were evaluated for the functional groups present in each NP form. In Fig. 2a, the 631 and 577 cm^− 1^ peaks may be associated with the Fe-O stretching vibration. In Fig. 2b, peaks at 1081, 800, 2015, and 1727 cm^− 1^ correspond to Si-O-Si stretching vibration, Si-O symmetric stretching vibration, isothiocyanate, and carbonyl group of lactone, respectively, indicating successful encapsulation of silica and a FITC layer onto the surface of Fe₃O₄ [55]. The peaks around 3500 and 3384 cm^− 1^ refer to N-H and B-O-H stretching, respectively, while sharp bands at 1720 and 1631 cm^− 1^ are related to C = C and C = O conjugation, respectively (Fig. 2c). In contrast, three prominent peaks at 1630, 1509, and 1380 cm^− 1^ correspond to the O-C = O, C = O, and B-O stretching bonds of bortezomib, respectively. Additionally, the bands at 537, 1715, and 1655 cm^− 1^ in Fig. 2c correspond to Fe₃O₄, ester, and amide groups, respectively, confirming the successful polymerization of PDA onto the surface of Fe₃O₄ nanoparticles.

### Microscopic analysis

**Fig. 3:**
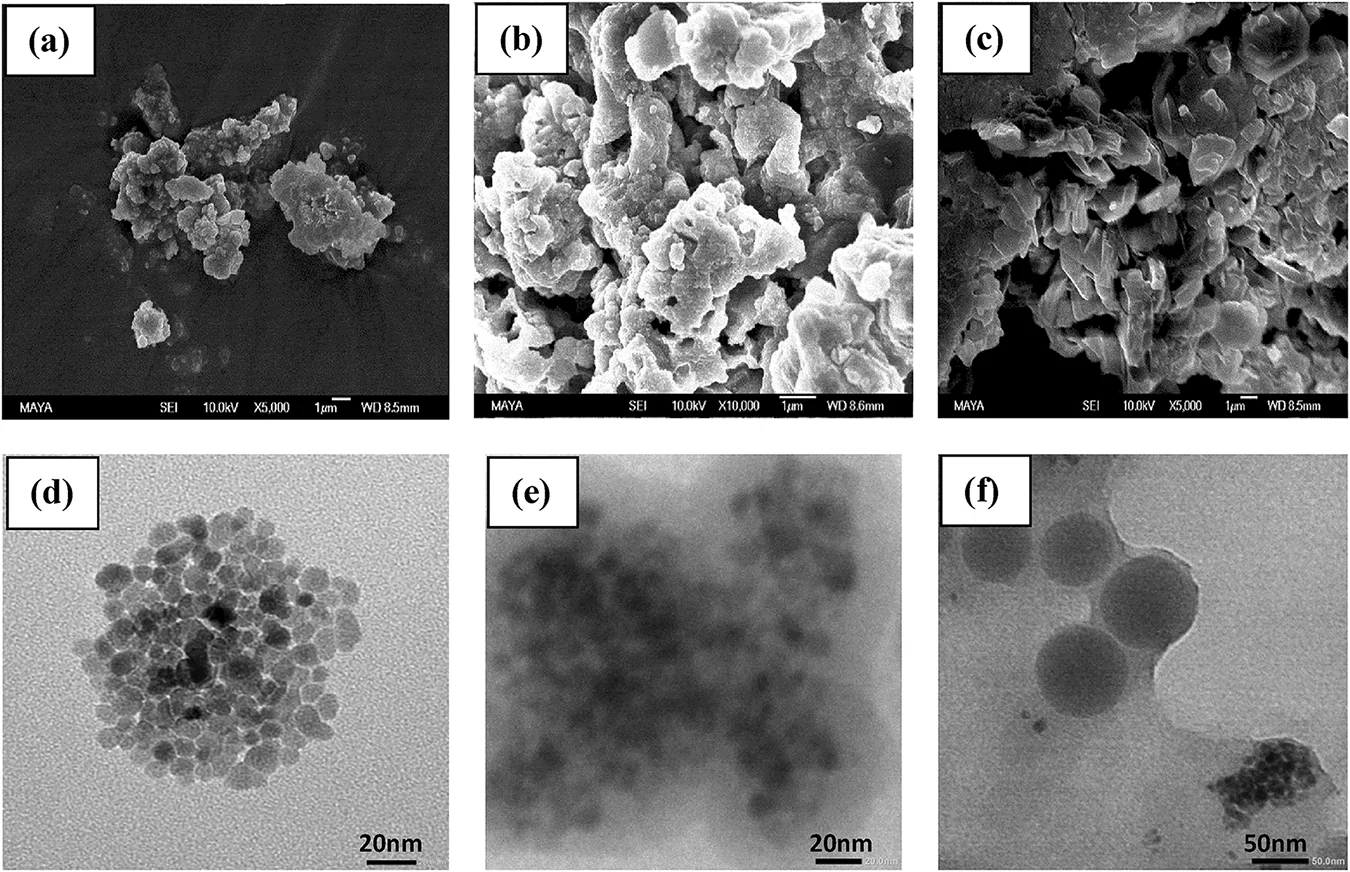
SEM and TEM images of synthesized materials: a,d) Fe₃O₄, b, e) core-shell, and c, f) FMMIP-BTZ

The size and morphology of the prepared nanocomposites were evaluated under vacuum in a dry state by SEM analysis (Fig. 3). The SEM image of Fe₃O₄ NPs reveals agglomerated clusters rather than well-dispersed individual particles (Fig. 3a). This agglomeration is common due to the high surface energy and magnetic interactions between iron nanoparticles [56]. The particle size appears to be in the sub-micron range, with individual particles measuring around 10–200 nm also given in DLS analysis (Fig. S1). The observed rough and irregular surface texture indicates a possible porous or polycrystalline nature, which can enhance adsorption properties. After that modification of Fe₃O₄ with SiO₂ and FITC provide a Fe₃O₄@SiO₂@FITC core-shell a highly porous and rough-textured surface, which is beneficial for increased surface area and enhanced adsorption capacity in chemical or biological interactions (Fig. 3b). The presence of nano-scale voids and channels within the silica coating suggest structure porosity, which could be advantageous for controlled drug release or selective adsorption. The layered and sheet-like structures observed in the SEM image (Fig. 3c) suggest the successful polymerization of dopamine, which leads to the formation of a polydopamine (PDA) coating on Fe₃O₄@SiO₂@FITC nanoparticles. The presence of aggregated and irregularly shaped particles indicates the interaction between BTZ and PDA-functionalized nanoparticles, which could result in enhanced drug loading and stability. Compared to uncoated Fe₃O₄@SiO₂@FITC core-shell nanoparticles, the surface appears more textured and rougher, which is typical for PDA coatings due to their adhesive and self-polymerizing properties.

TEM images of Fe₃O₄ nanoparticles, core-shell, and FMMIPs are shown in Fig. 3d and e, and 3f, respectively. Fe₃O₄ nanoparticles modified with a citrate group (bar scale of 20 nm) give approximately 10 nm particle size and are presented in Fig. 3d. This image represented a broad size distribution, indicating some degree of polydispersity. The Fe₃O₄ NPs, which appeared in spherical clusters, easily combined with TEOS, PDA, and BTZ due to their small diameter. The Fe₃O₄ silica core-shell shows some outer coverage due to the coating of both TEOS and FITC which increase the particle size up to ± 29.14 nm (Fig. 3e). The low contrast in the outer layer suggests that TEOS (Tetraethyl Orthosilicate) derived silica has successfully coated the iron core. Figure 3f indicates the increased particle size approximately 50–60 nm of FMMIP-BTZ due to polymerization of dopamine and binding of BTZ (bar scale at 50 nm). To support the SEM and TEM figures, DLS analysis is given in the supporting information Fig. S1.

### Magnetic study

**Fig. 4:**
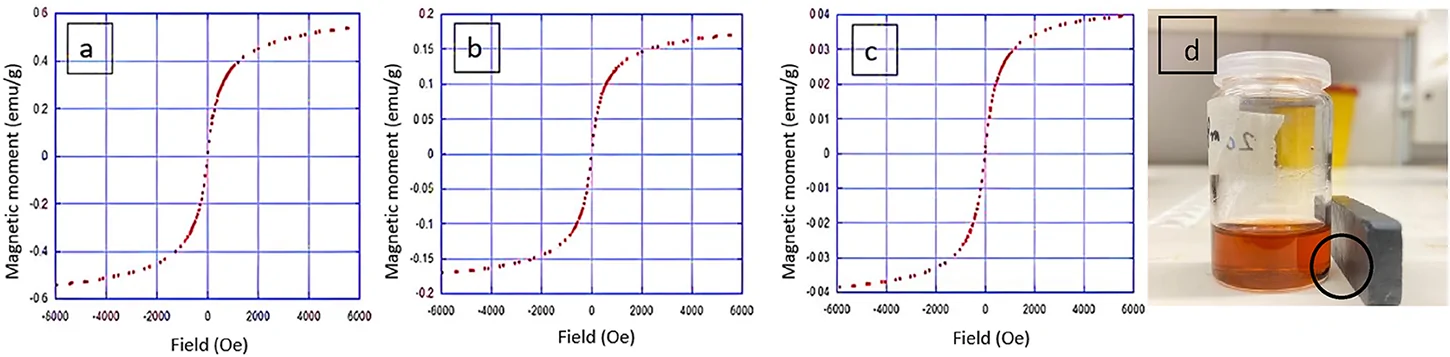
Vibrating sample magnetometer hysteresis loops of synthesized materials: (a) Fe₃O₄, (b) core-shell (c) FMMIP-BTZ (d) Photographic accumulation of FMMIP under the influence of an external magnetic field

A vibrating sample magnetometer (VSM) was used to examine the magnetic saturation (Ms) properties of Fe₃O₄ NPs, Fe₃O₄ @SiO₂@FITC core-shell, and Fe₃O₄@SiO₂@FITC@PDA-BTZ, as in Fig. 4a-c and Table S1. Fe₃O₄ nanoparticles free of any coatings or modifications exhibited the highest magnetization due to their purely magnetic composition (Fig. 4a). The amalgamation of any non-magnetic material such as SiO₂ or FITC together with magnetic material resulted in reduction in overall magnetism (Fig. 4b). A further decrease in the magnetization of Fe₃O₄@SiO₂@FITC core-shell was observed after the incorporation of polydopamine and BTZ (Fig. 4c). After adding various non-magnetic layers to Fe₃O₄ NPs, the magnetic properties of the Fe₃O₄ were significantly reduced [24]. Quantitatively, Ms fell from 0.48 emu/g (Fe₃O₄) to 0.17 emu/g (core-shell, a 64.6% reduction) and to 0.04 emu/g for FMMIP-BTZ (91% overall reduction; Table S1). This decrease is consistent with the increase in non-magnetic shell thickness observed by TEM, where particle diameter grows from approx.10 nm (Fe₃O₄) to 50–60 nm (FMMIP-BTZ). A photograph of FMMIP collected at the vial wall under an external magnetic field is given below to demonstrate practical separability despite the low Ms value (Fig. 4d).

d.

### TGA analysis

**Fig. 5:**
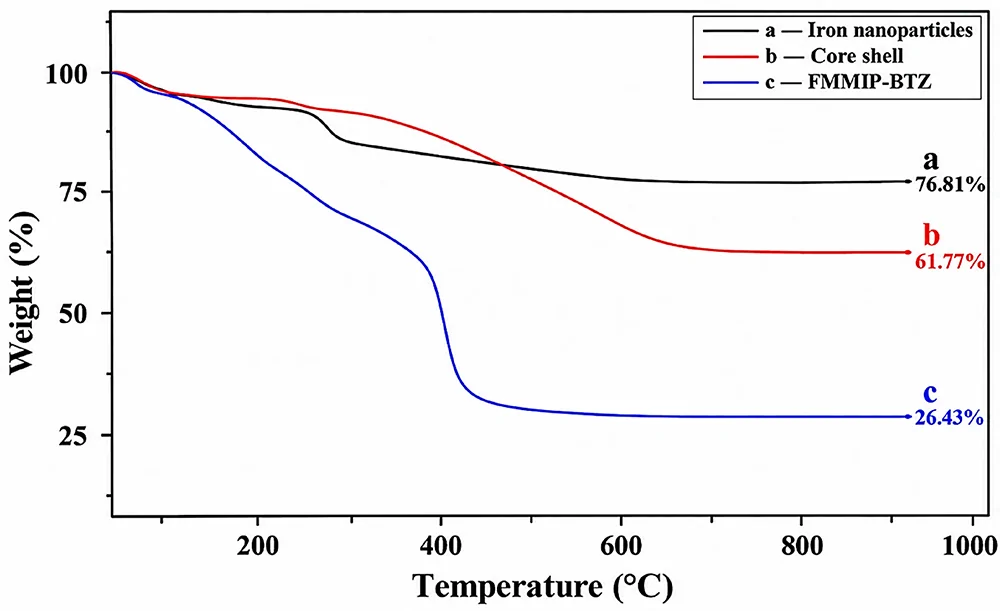
TGA analysis of synthesized materials: (**a**) Fe₃O₄, (**b**) core-shell, and (**c**) FMMIP-BTZ

TGA analysis was used to assess the amount of magnetite in the synthesized nanomaterials and the cross-linking yield of the imprinted polymer coatings. TGA curves for Fe₃O₄, Fe₃O₄ @SiO₂@FITC core-shell, and FMMIP-BTZ are shown in Fig. 5 (a-c). A thermogram of Fe₃O₄ NPs (Fig. 5a) revealed a significant weight loss (13%) at around 280 °C due to the removal of the citrate group (present due to the use of Trisodium citrate during Fe₃O₄ NPs synthesis) and moisture. In contrast, weight loss in Fe₃O₄ NPs at 300–500 °C was attributed to labile oxygen breakdown. A weight loss of roughly 38% in the thermogram (Fig. 5b) indicated the degradation of grafted silica and FITC on the surface of the Fe₃O₄@SiO₂@FITC core-shell. In the thermogram of FMMIP-BTZ (Fig. 5c), weight losses of approximately 38% and 36% were observed below 400 °C and between 400 and 600 °C, respectively. This significant weight loss is attributed to the combined effects of many processes, such as loss of adsorbed water and decomposition of FMMIP-BTZ nanocomposites and bortezomib.

### XRD analysis

**Fig. 6:**
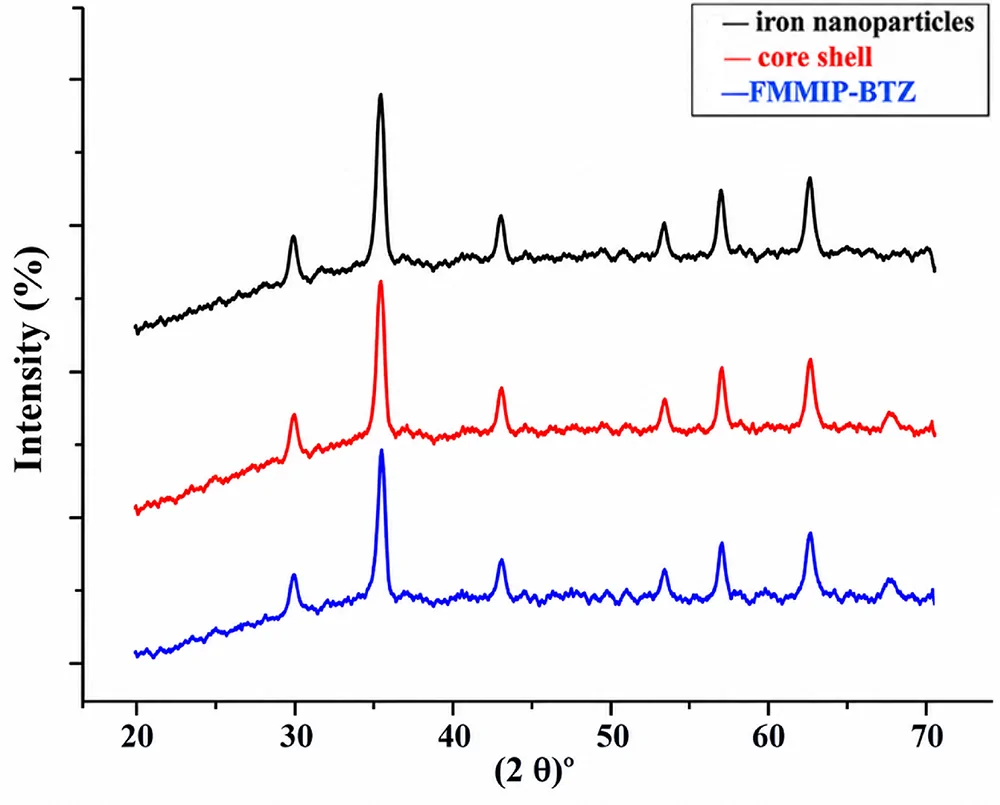
XRD patterns of Fe_3_O_4_, core shell, and FMMIP-BTZ

X-ray diffraction analysis was used to determine drug carrier crystallinity and the structural properties of synthesized Fe₃O₄, core shells, and FMMIP-BTZ Fig. 6. Diffusivity peaks in the 2θ region of 10° to 75° (30°, 35°, 43.5°, 53.6°, 56.0°, and 63°) were consistent with the characteristic spectrum of Fe₃O₄ NPs and their core-shell. These peaks had crystalline planes of (2 2 0), (3 1 1), (4 0 0), (4 2 2), (5 1 1), and (4 4 0), respectively. The XRD patterns of synthesized FMMIP-BTZ showed broad peaks from 12° to 26°, supporting the development of an amorphous polymeric layer on the surface of Fe₃O₄ NPs. Strong refractive peaks in the 30° to 75° region were also consistent with the Fe₃O₄ pattern. The similarity of the Fe₃O₄, core-shell, and FMMIP-BTZ patterns suggests the preservation of the crystallography of magnetic NPs during the development of FMMIP on the Fe₃O₄ surface. The gradual reduction in peak intensity of synthesized materials indicates successful silica, FITC, and PDA coating on the surface of Fe₃O₄ NPs and binding with BTZ.

### Fluorescence study of FMMIPs

**Fig. 7:**
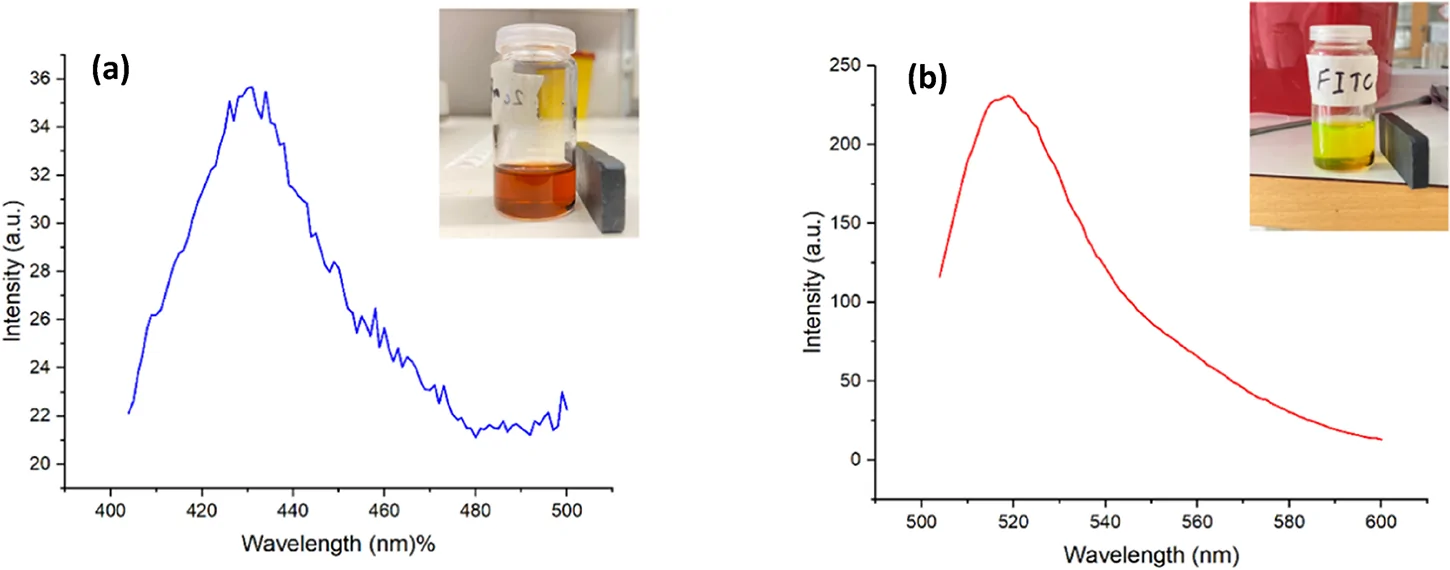
Image of fluorescence color before (**a**) and after (**b**) releasing of drug Fluorescence value of bortezomib drug (**a**) and FITC (**b**)

It is important to mention that two different fluorescent layers are present on FMMIP-BTZ: (1) due to BTZ and (2) due to FITC. The fluorescence of FMMIP-BTZ was determined after the release of BTZ and also after the degradation of the polydopamine layer on the remaining core-shell nanomaterial. To observe the fluorescence, 25 mg of FMMIP-BTZ was added into 20 mL of phosphate buffer solution (PBS) and dimethyl sulfoxide (DMSO) at a 6:4 ratio at neutral pH. After 20 min, the solution colour changed to orange-brown, as shown in Fig. 7a, and the remaining insoluble magnetic particles (MMIPs) had settled. The insoluble magnetic MMIP were separated from the orange-brown solution and dried in a vacuum desiccator. The fluorescence of BTZ was investigated in the orange-brown solution using a fluorimeter with excitation at 360 nm and emission at 460 nm. The remaining magnetic MMIP were insoluble in water and ethanol due to strong covalent bonding between TEOS-coated iron nanoparticles and FITC.

For measurement of the second layer of fluorescence, the degradation of polydopamine was induced by adding insoluble MMIPs into a buffer solution of pH 13 under magnetic stirring at 60 °C [57]. After 48 h, the insoluble particles were separated from the solution under a magnetic field and dried in a vacuum desiccator. The dried particles were put in a solution of DMSO and placed in a shaker for 24 h. A bright yellow color appeared due to FITC (Fig. 7b), and particles were assessed for fluorescence using a fluorimeter at 525 nm. The steady-state photoluminescence (PL) emission spectra of the released BTZ and the unmasked FITC layer are shown in Fig. 7a and b respectively. Under excitation at 360 nm, BTZ exhibited a PL emission maximum at 437 nm (intensity ~ 36.3 a.u.), while FITC, following degradation of the polydopamine layer, exhibited a PL emission maximum at 513 nm (intensity ~ 227 a.u.). These measurements represent steady-state PL emission characterization; fluorescence decay constants and lifetimes were not determined in this study, as such parameters require time-correlated single-photon counting (TCSPC) or an equivalent time-resolved fluorometer, which was outside the scope of the present work.

### Reproducibility measurements for release and adsorption of BTZ

To investigate the release and absorption of BTZ, 170 mg of synthesized FMMIP-BTZ were added to 20 mL of DMSO and PBS solution at a ratio of 4:6. The solution became orange-brown due to the solubility of fluorescent BTZ. The insoluble MMIPs in the above solution were separated by an EMF and dried in a vacuum desiccator. The rebinding of BTZ on separated dried MMIP particles was executed by immersing 125 mg of separated dried MMIP particles in 100 mL of 1 mmol (38.4 mg/100 mL) BTZ solution for 5 h at 25 °C to attain adsorption equilibrium. Sink conditions were maintained by ensuring that the concentration of released bortezomib remained below 10% of its saturation solubility in the receptor medium. The receptor volume was sufficient to prevent saturation during the entire release period. Afterwards, BTZ rebound particles (FMMIP-BTZ) were separated under an EMF, washed with deionized water, dried under a vacuum for 16 h, and weighed again. The mass of examined particles increased from 125 to 151.6 mg due to rebinding of BTZ, corresponding to 26.6 mg of BTZ bound per 125 mg of MMIP. The rebinding solution offered 38.4 mg of BTZ in 100 mL of a 1.0 mM of BTZ solution resulting in an encapsulation efficiency of 69.3% and a loading capacity of 17.5%. Both the encapsulation efficiency and loading capacity were calculated using Eqs. 2 and 3. Different FMMIP solutions with 100–500 ppm concentrations were prepared in five measuring flasks to execute the batch-release experiments. In each solution, 20 mL of DMSO and PBS solution were added in the ratio of 4:6 at pH = 7.47 and 25 °C. The cumulative drug release was calculated according to Eq. 4 [58]:

2 $$\begin{array}{ll}{\rm{Encapsulation}}\,{\rm{Efficiency}}\,({\rm{EE}}\%)=&\frac{\mathrm{m}\mathrm{a}\mathrm{s}\mathrm{s}\:\mathrm{o}\mathrm{f}\:\mathrm{B}\mathrm{T}\mathrm{Z}\:\mathrm{l}\mathrm{o}\mathrm{a}\mathrm{d}\mathrm{e}\mathrm{d}}{\mathrm{m}\mathrm{a}\mathrm{s}\mathrm{s}\:\mathrm{o}\mathrm{f}\:\mathrm{B}\mathrm{T}\mathrm{Z}\:\mathrm{i}\mathrm{n}\mathrm{i}\mathrm{t}\mathrm{i}\mathrm{a}\mathrm{l}\mathrm{l}\mathrm{y}\:\mathrm{a}\mathrm{d}\mathrm{d}\mathrm{e}\mathrm{d}}\times\:100\\{\rm{Encapsulation}}\,{\rm{Efficiency}}\,({\rm{EE}}\%)=&\frac{26.6}{38.4}\times\:100=69.3\%\end{array}$$

3 $$\begin{array}{ll}{\rm{Loading}}{\rm{Capacity}}({\rm{LC}}\%)=&\frac{\mathrm{m}\mathrm{a}\mathrm{s}\mathrm{s}\:\mathrm{o}\mathrm{f}\:\mathrm{B}\mathrm{T}\mathrm{Z}\:\mathrm{l}\mathrm{o}\mathrm{a}\mathrm{d}\mathrm{e}\mathrm{d}}{\mathrm{m}\mathrm{a}\mathrm{s}\mathrm{s}\:\mathrm{o}\mathrm{f}\:\mathrm{l}\mathrm{o}\mathrm{a}\mathrm{d}\mathrm{e}\mathrm{d}\:\mathrm{c}\mathrm{a}\mathrm{r}\mathrm{r}\mathrm{i}\mathrm{e}\mathrm{r}}\times\:100\\{\rm{Loading}}\,{\rm{Capacity}}({\rm{LC}}\%) =& \frac{26.6}{151.6}\times\:100=17.5\%\end{array}$$

4 $$\:Q\left(\%\right)=\frac{{C}_{n}{V}_{n}+{V}_{i}\:{\sum\:}_{i=1}^{n-1}{C}_{i}}{m}$$

where Q is the percent of the cumulative release of BTZ; C_n_ and V_n_ refer to the concentration and volume of the release media, respectively, C_i_ and V_i_ are the concentration of BTZ and volume of each sample, respectively; and m is the mass of BTZ initially loaded onto the FMMIP-BTZ sample used in the release experiment (rather than the total mass of the FMMIP-BTZ carrier).

The absorbance of each orange-brown solution obtained after the release of BTZ at room temperature at 390 nm was studied using a UV-vis spectrophotometer to determine the concentration of BTZ released from FMMIP-BTZ. Fig. S2 demonstrates that the highest absorbance corresponds to 500 ppm orange-brown solution and the lowest absorbance to the DMSO reference without BTZ. It can be inferred from the absorption spectra that the absorbance is directly related to the concentration of FMMIP-BTZ, which is proportional to the amount of drug released from the imprinted polymer. Fluorescent MMIPs demonstrated excellent release properties, with 21.43 mg (0.0558 mmol) of BTZ successfully released from the FMMIP-BTZ complex, corresponding to a cumulative release of 80.56%. BTZ release from polydopamine-coated MMIP occurred because weak interactions destabilized in DMSO and PBS, and it can be separated under an EMF at room temperature [59, 60]. The release capacity of the anticancer drug was calculated from Eq. (4). The results revealed excellent release properties of synthesized material and quick identification.

Non-imprinted polymers were loaded with the same quantity of BTZ as molecularly imprinted polymers by following the same procedure used for FMMIP-BTZ. NIPs with different BTZ concentrations ranging from 100 to 500 ppm were prepared. The absorbance of each solution was measured with a UV-vis spectrophotometer using the same procedure adopted for FMMIP-BTZ. It can be observed from the absorption spectrum (Fig. S3) that non-selective binding of BTZ occurs on NIPs due to the presence of O-H, N-H, and C = O groups.

### pH-dependent release of BTZ from FMMIPs

**Fig. 8:**
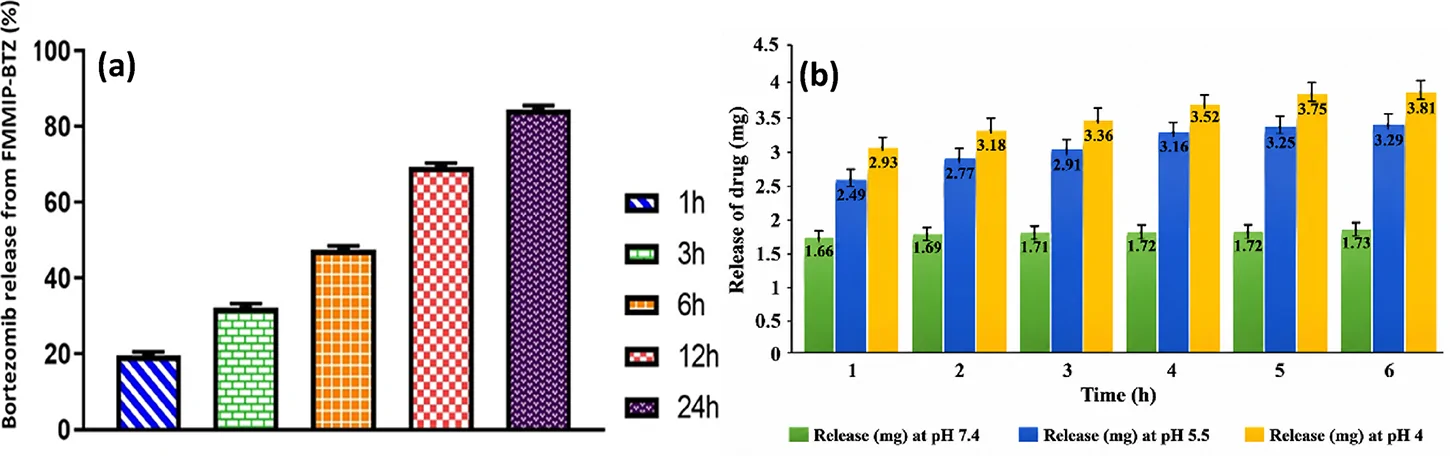
The cumulative amount of percentage release of BTZ from FMMIP-BTZ in vitro diffusion studies (**a**) and at pH 4, 5.5 and 7.4 (**b**)

The cumulative amount of BTZ was released from in vitro diffusion studies of FMMIP-BTZ formulation as given in Fig. 8a. The releasing results were calculated after 24 h and percentage release of BTZ from FMMIP-BTZ was observed as 84.50 ± 1%. To evaluate the effect of pH on controlled BTZ release from Fe₃O₄ @SiO₂@FITC@PDA-BTZ nanomaterial in 1 to 6 h, the release patterns of BTZ were compared using three solutions at pH values of 4, 5.5, and 7.4. In the solution with pH 4, BTZ release was initially rapid and became slower with time, eventually slowing to minimal release in the last hour. At pH 7.4, the release of BTZ from the nanocarrier was minimal and remained fairly consistent throughout the stipulated time. The remaining nanoparticles (MMIPs) obtained after the release of BTZ were washed and placed in a vacuum desiccator for two days. The nanoparticles were weighed again, and percentage release of BTZ was compared among pH values (Table S2). As we increased the duration of time from 1 to 6 h, the release of BTZ reached a maximum of 6 h, as shown in Fig. 8b.

### Primary dermal irritation index

**Fig. 9:**
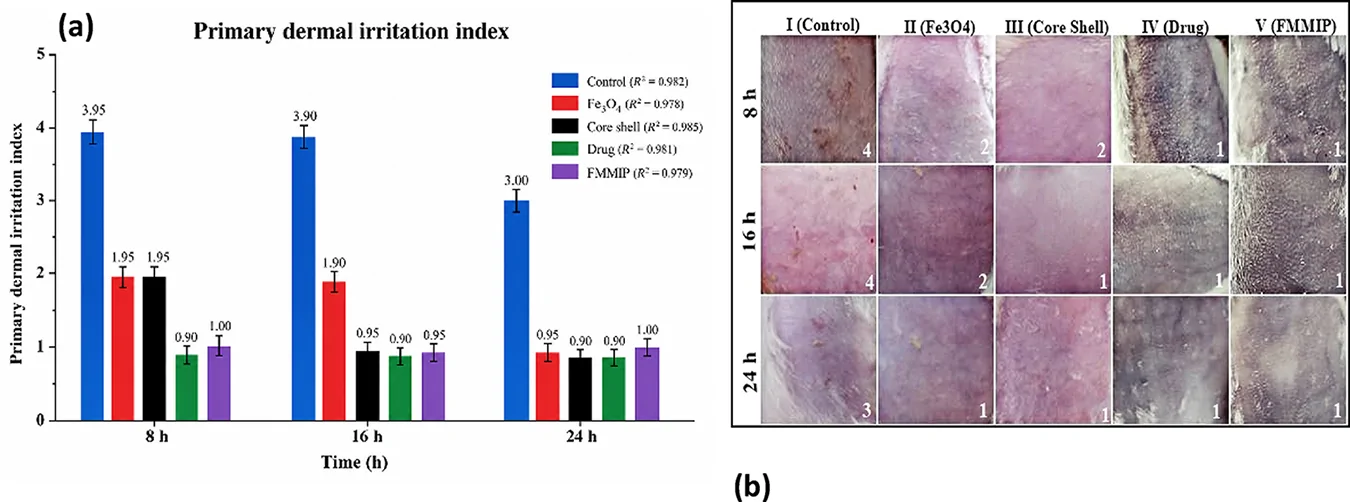
Primary dermal irritation index in vivo study of bortezomib released from FMMIP-BTZ formulation

Figure 9a illustrates that distinct changes in the skin were assessed using the mean erythema values: 1 for no erythema, 2 for light pink, 3 for dark pink, and 4 for dark red. The Fig. 9a ‘shows that rats exposed to ammonia experienced severe irritation, evidenced by dark red discoloration, while the other four groups treated with Fe₃O₄, core-shell, the test drug, and FMMIP did not exhibit dark red or dark pink signs of erythema. This indicates that the treated rats did not develop skin irritation. The findings demonstrate visible skin reactions, with only the ammonia-treated group showing severe irritation, as given in Fig. 9b. In contrast, the other groups showed no signs of erythema, confirming the absence of skin irritation.

### Molecular docking results

Molecular docking the most widely used approach in the field of drug discovery and many drug candidates have been identified by molecular docking studies [61, 62]. The molecular docking studies provide insights of molecular interaction between the nanoparticles and the macromolecular drug targets [63].

**Fig. 10:**
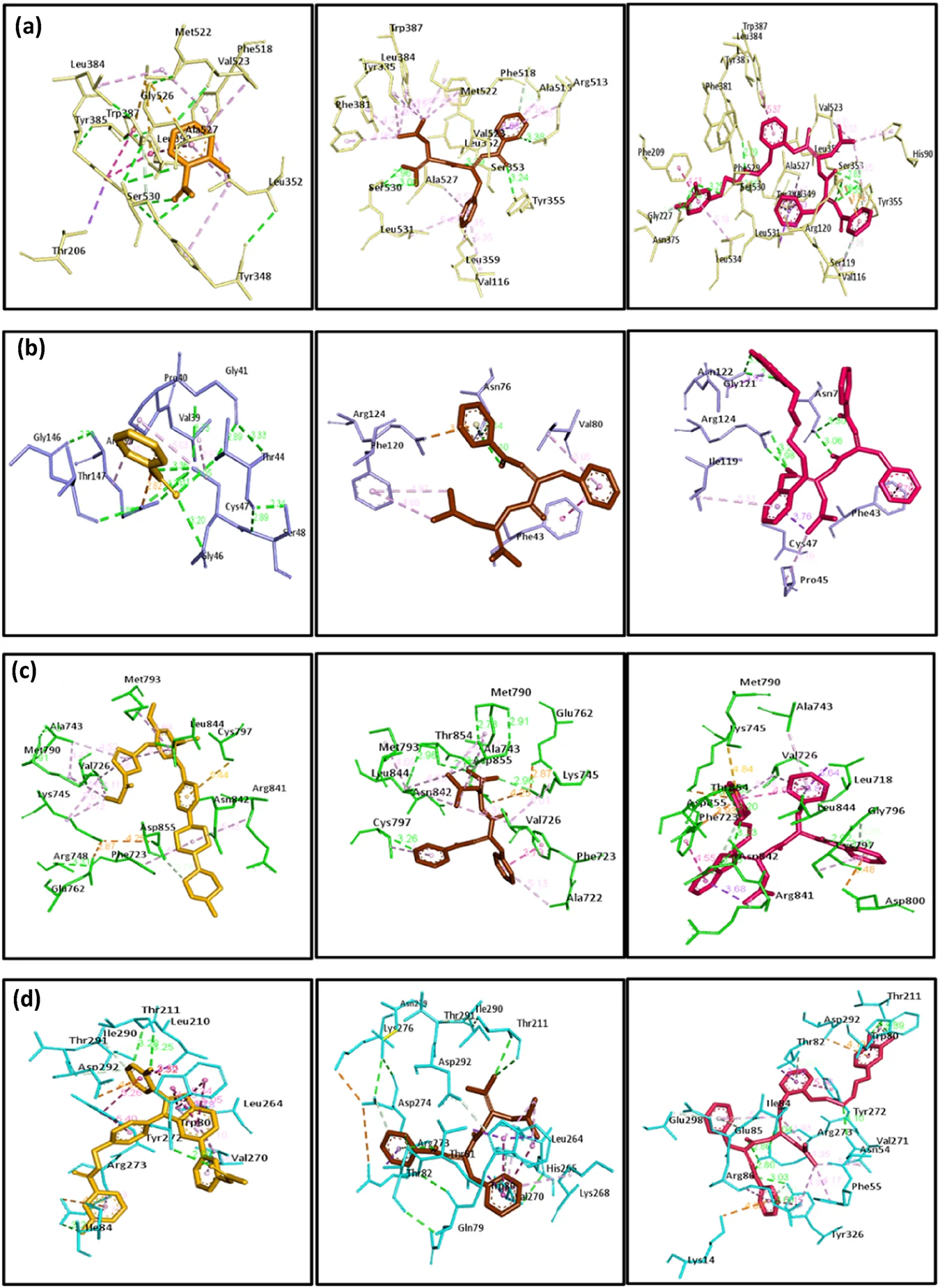
Molecular docking studies against different drug targets. The three-dimensional interactions of ligands into the active site of (**a**) human cyclooxegenase 2 (COX-2), (**b**) human peroxiredoxin5 (PDRX-5), (**c**) human EGFR, (**d**) human AKT1are shown. The respective standard inhibitors (**a**) salicylic acid (**b**) benzoic acid (**c**) naquotinib (**d**) AKT inhibitor 12j are shown by orange colour sticks. The bortezomib is shown by brown colour while FMMIP-BTZ is shown by pink color

The enzyme cyclooxegenase-2 (COX-2) has been found to involved in inflammation as well as different types of cancer [64]. It is upregulated under inflammatory response of body and increase the production of prostaglandins leading towards skin redness, itching and erythema [65, 66]. The three-dimensional structure of COX-2 in complex with its inhibitor salicylic acid (PDB ID 5F1A) was used in this study for assessment of erythema. All the ligands were docked into the active site of COX-2 and their results have been shown in Fig. 10a. The ligands NH_3_ and Fe₃O₄ did not show any significant interaction with COX-2 and exhibited very poor docking scores. Core shell, bortezomib and FMMIP showed higher docking scores and good interaction with COX-2. Particularly the nanocomposite showed the highest number of hydrogen bonds and interaction with the amino acid residues (Arg 120, Ser 530, Tyr 355, His 90) of active site pocket of COX-2.

Similarly, the enzyme peroxiredoxin-5 (PRDX5) plays a pivotal role in providing defense against oxidative damage during inflammation and protects skin from irritation [67]. Figure 10b shows the interaction of ligands obtained after molecular docking studies on PRDX5. As compared to COX-2 all the ligands showed lesser docking scores when docked into PRDX5. The docking protocol was validated by docking benzoic acid into the active site pocket of PRDX5. The best docking score was given by molecular docking of FMMIP among all the ligands which is even better than the reference molecule benzoic acid. Moreover, only FMMIP showed interaction with the main catalytic residue Cys 47. Which shows that FMMIP showed the most favorable predicted docking score among the tested models.

Epidermal growth factor receptor (EGFR) is activated on various external stimuli, including UV radiation and is reported to be involved in skin irritation, carcinogenic response of the skin; its EGFR activation contributes to skin inflammatory responses [68–70]. The interaction of compounds with the active site residues of EGFR has been depicted in Fig. 10c. The main catalytic residues of EFGR include Cys 797, Gln 791, Met 790, Met 793, and Leu 844. The binding affinity of FMMIP is almost similar to that shown by the reported inhibitor. The most promising interaction with active site residues of EFGR has been shown by the FMMIP nanocomposite with the highest number of hydrogen bonds.

Akt1 a serine/threonine kinase that plays an important role in cell proliferation and angiogenesis [71]. AKT is majorly involved in skin homeostasis and a variety of skin disorders [72]. The inhibition of AKT will regulate the over activation of ACKT which would suppress the inflammatory response. In molecular docking studies against AKT1 the FMMIP showed the best docking score among all ligands and the most significant receptor-ligand interaction established by six hydrogen bonds with the key residues of AKT1 including Thr 211, Asn 54 and Trp 80. The interaction of ligands with AKT1 obtained by molecular docking studies have been shown in Fig. 10d.

The results of molecular docking studies in Table 1 indicate that FMMIP-BTZ showed favorable computational interactions with inflammation-associated protein targets, suggesting possible molecular interactions that require experimental validation. Moreover, NH_3_ and Fe₃O₄ did not show any interaction with the binding site residues of all these four proteins and also gave extremely low docking scores.

**Table 1:** The results of molecular docking studies of all ligands against various drug targets

Sr. No.	Protein	Binding Affinity (kcal/mol) Reference Molecule	Binding Affinity (kcal/mol) NH_3_	Binding Affinity (kcal/mol) Fe₃O₄	Binding Affinity (kcal/mol) Core shell	Binding Affinity (kcal/mol) Bortezomib	Binding Affinity (kcal/mol) FMMIP
1.	COX-2	(Salicylic acid) -6.0	-1.6	-4.3	-7.6	-8.7	-8.0
2.	PRDX5	(Benzoic acid) -5.0	-1.5	-3.2	-6.4	-6.1	-6.5
3.	EGFR	(Naquotinib) -7.9	-1.4	-4.0	-7.3	-6.5	-7.1
4.	AKT1	(Inhibitor 12j) -12.2	-1.3	-3.6	-10.4	-9.4	-11.0

### Molecular dynamics simulation of AKT1–bortezomib complex

The dynamic stability of the AKT1–bortezomib complex was evaluated by a 100 ns all-atom MD simulation using OpenMM 8.0. AKT1 (PDB ID: 4EJN) was selected based on the molecular docking results, where FMMIP-BTZ showed the highest predicted affinity for AKT1 (− 11.0 kcal/mol), compared with COX-2 (− 8.0 kcal/mol), EGFR (− 7.1 kcal/mol), and PRDX5 (− 6.5 kcal/mol). Because FMMIP-BTZ is a multicomponent nanocomposite, bortezomib was used as the representative ligand for MD simulation, as it is the pharmacologically active template and has a well-defined three-dimensional structure suitable for force-field parameterization.

**Fig. 11:**
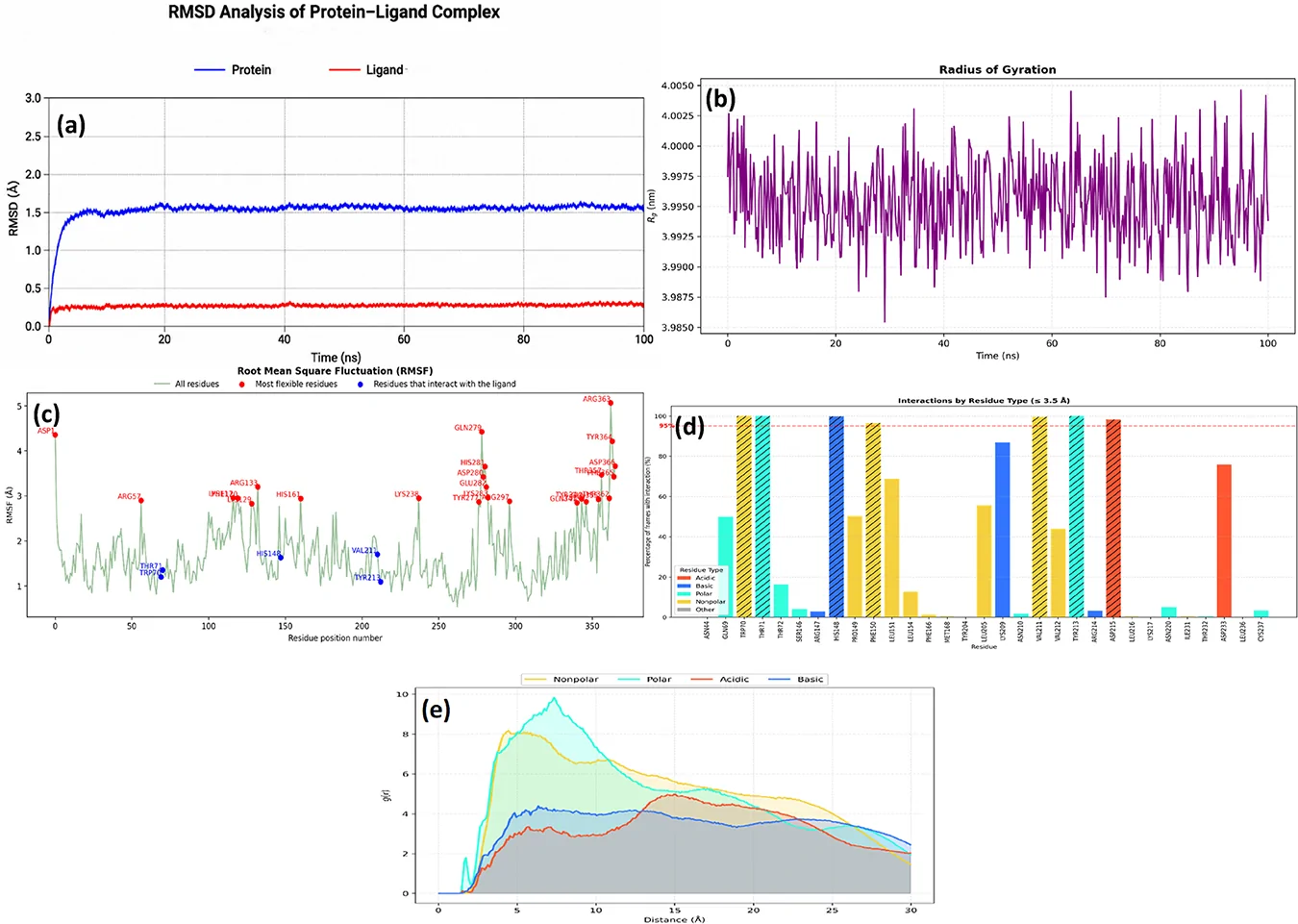
Molecular dynamics analysis of the AKT1–bortezomib complex during the 100 ns simulation: (**a**) Root mean square deviation (RMSD) of the AKT1 protein backbone Cα atoms and bortezomib (**b**) Radius of gyration (Rg) of AKT1–bortezomib complex (**c**) Per-residue root mean square fluctuation (RMSF) of AKT1 Cα atoms of the AKT1–bortezomib complex (**d**) Number of intermolecular hydrogen bonds between bortezomib and AKT1 (**e**) Solvent-accessible surface area (SASA) profiles of the apo AKT1 and AKT1–bortezomib complexes, showing the contributions of nonpolar, polar, acidic, and basic residues. The combined profiles indicate preservation of the docked bortezomib binding pose, limited flexibility at the principal binding-site residues, and maintenance of the overall structural integrity of AKT1 throughout the simulation

The RMSD profiles showed that the AKT1 backbone reached equilibrium within approximately 10–15 ns and remained stable thereafter (Fig. 11a). The ligand RMSD also remained low throughout the trajectory, indicating retention of the docked bortezomib binding pose. These results support the stability of the predicted AKT1–bortezomib complex under explicit-solvent MD conditions.

RMSF analysis showed relatively low fluctuations for the key binding-site residues Thr211, Asn54, and Trp80, whereas higher fluctuations were mainly observed in solvent-exposed loops and terminal regions (Fig. 11c). This pattern indicates that bortezomib binding does not induce substantial flexibility at the predicted interaction site.

The radius of gyration remained essentially stable throughout the 100 ns trajectory (Fig. 11b), indicating preservation of the overall compact structure of AKT1 without major conformational expansion.

Intermolecular hydrogen-bond analysis further supported persistence of the protein–ligand interactions, particularly involving Thr211, Asn54, and Trp80 (Fig. 11d). The continued presence of these interactions throughout the simulation is consistent with the experimentally relevant binding stability predicted from docking.

Intermolecular hydrogen-bond analysis further supported persistence of the protein–ligand interactions, particularly involving Thr211, Asn54, and Trp80 (Fig. 11d). The continued presence of these interactions throughout the simulation is consistent with the experimentally relevant binding stability predicted from docking.

The Solvent-accessible surface area (SASA) profile remained stable with a slight reduction relative to the apo AKT1 (Fig. 11e), consistent with limited changes in solvent exposure upon bortezomib binding. Overall, the RMSD, Rg, RMSF, hydrogen-bond, and SASA analyses consistently indicate that the docked bortezomib pose is maintained and that AKT1 retains its overall structural integrity during the 100 ns simulation. These findings provide dynamic support for the docking-based interaction of the bortezomib component of FMMIP-BTZ with AKT1.

### Release kinetics study

**Fig. 12:**
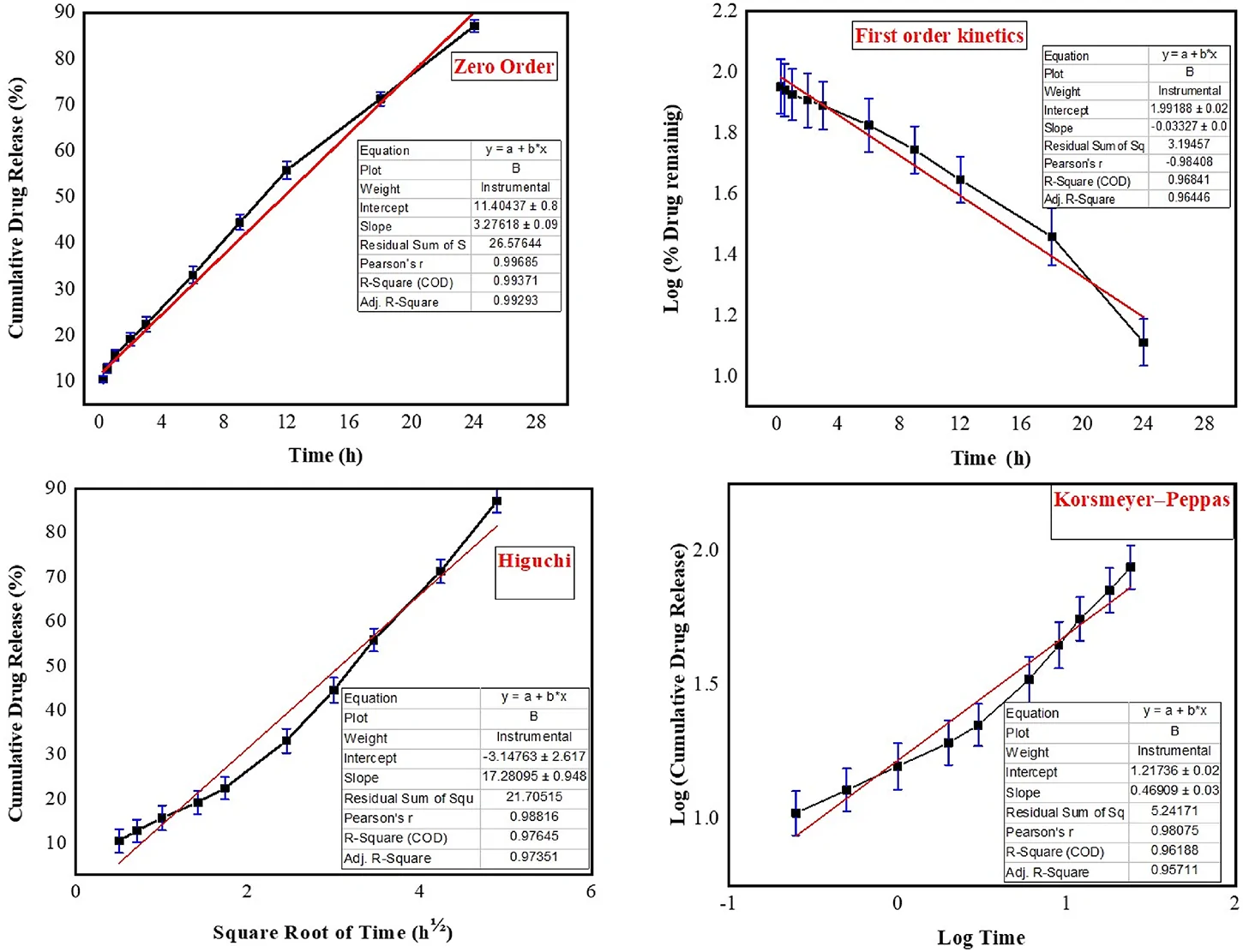
Drug release kinetic modeling of bortezomib (BTZ) released from the synthesized Fe₃O₄@SiO₂@FITC@PDA-BTZ fluorescent magnetic molecularly imprinted polymer (FMMIP-BTZ). The experimental release data were fitted to (a) Zero-order, (b) First-order, (c) Higuchi, and (d) Korsmeyer–Peppas kinetic models to elucidate the release mechanism of BTZ from the nanocarrier. The correlation coefficients (R²) obtained from each model were compared to determine the best-fitting kinetic model and the predominant drug release mechanism

The in vitro release data of BTZ from the synthesized FMMIP-BTZ were fitted to zero-order, first-order, Higuchi, and Korsmeyer–Peppas kinetic models to evaluate the release behavior and identify the model that best described the experimental data (Fig. 12). The corresponding regression parameters and correlation coefficients were compared to determine the most appropriate kinetic model.

The zero-order model provided the best fit to the experimental release data, with a regression equation of Qₜ = 11.404 + 3.276t, where Qₜ represents the cumulative percentage of BTZ released at time t. The calculated zero-order release rate constant (k₀) was 3.276% h⁻¹, with R² = 0.9937 and adjusted R² = 0.9929. The high linearity indicates that BTZ was released at an approximately constant rate over the investigated period.

The Higuchi model also showed a relatively good fit, yielding a Higuchi release constant (kH) of 17.281, with R² = 0.9765 and adjusted R² = 0.9735. The comparatively lower R² than that of the zero-order model suggests that diffusion contributed substantially to BTZ release but did not completely describe the overall release profile.

The first-order model yielded the regression equation log₁₀(100 − Qₜ) = 1.99188 − 0.03327t, with R² = 0.9684 and adjusted R² = 0.9645. Based on the conventional first-order expression log₁₀(C₀/Cₜ) = k₁t/2.303, the corresponding release rate constant was calculated as k₁ = 0.0766 h⁻¹. The lower correlation coefficient compared with the zero-order and Higuchi models indicates that the release profile was less well described by a concentration-dependent first-order process.

The Korsmeyer–Peppas model produced a kinetic constant (k) of 1.217 and a release exponent (n) of 0.469, with R² = 0.9619 and adjusted R² = 0.9571. Among the investigated models, this model showed the lowest R², indicating a comparatively poorer fit to the complete release profile. The release exponent provides information regarding the contribution of diffusion and other transport processes; however, its mechanistic interpretation should be made with consideration of the geometry and assumptions of the particular release system.

Overall, the goodness-of-fit followed the order: Zero-order > Higuchi > First-order > Korsmeyer–Peppas. The superior fit of the zero-order model indicates that the overall BTZ release from FMMIP-BTZ was best described by an approximately constant release rate over the investigated period, supporting the controlled and sustained release characteristics of the developed nanocarrier.

### Imprinting factor

**Fig. 13:**
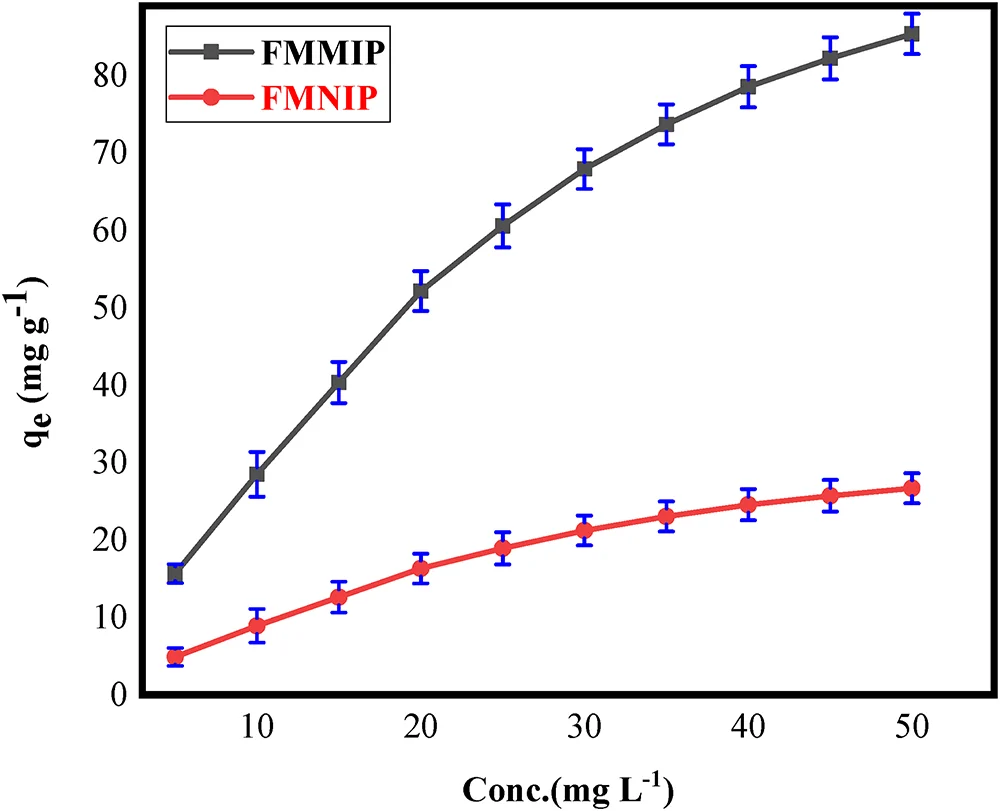
Comparison of the binding capacities of FMMIP vs. FMNIP (*n* = 5)

To evaluate the selectivity and molecular recognition ability of the synthesized fluorescent magnetic molecularly imprinted polymer (FMMIP) for bortezomib (BTZ), the imprinting factor (IF) was calculated. As shown in Fig. 13, FMMIP showed a much higher adsorption capacity than the corresponding fluorescent magnetic non-imprinted polymer (FMNIP), which confirmed the successful formation of specific recognition sites complementary to BTZ. The adsorption of FMNIP is due to nonspecific interactions on the polymer surface. The equilibrium adsorption capacities of FMMIP and FMNIP were 85.34 mg g⁻¹ and 26.67 mg g⁻¹, respectively. The selectivity of the imprinted polymer was assessed by using the imprinting factor, which is calculated as the ratio of the adsorption capacities of FMMIP and FMNIP. An imprinting factor greater than 1 is generally considered indicative of successful molecular imprinting [73]. The obtained IF value of 3.20 in the present study indicated the strong affinity and high selectivity of FMMIP towards BTZ. This value is comparable to the previously reported MIP systems, such as IF values of 2.02 for lufenuron [74], 2.64 for fipronil [75], and 1.64 for pirimiphos-methyl [76]. These comparisons suggest that the synthesized FMMIP presents excellent molecular recognition performance and can effectively create BTZ-specific binding cavities within the polymer matrix. The results obtained validate the effectiveness of the imprinting strategy and emphasize the potential of the developed FMMIP as a promising platform for selective drug loading, recognition and controlled drug-delivery applications.

### Comparative study of FMMIP nanocomposite with previously published materials

To reduce the non-specific adsorption of drugs, it is important to consider a number of options, such as increasing the concentration of drugs in the intracellular medium of cancer cells, lowering their toxic effects in healthy cells, and extending the half-life of the drugs in blood serum through the development of novel drug delivery systems with unique traits. An effective DDS must comprise components resistant to erosive forces and offer enteric solubility and hydrophobicity. Furthermore, it should be biocompatible, biodegradable, and sustainable.

The performance of the newly synthesized FMMIP-BTZ nanocomposite was compared with materials from previous studies in terms of release capacities and Preliminary dermal compatibility (Table 2). Most DDSs are bio-compatible, though only a few of them are biodegradable. To ensure the drug’s availability to the cells, its releasing capacity in the stipulated time is an important parameter governing the performance of a typical DDS. The data summarized in Table 2 indicate that most DDSs have release capacities less than 70% in 6 h. For instance, the controlled release of sunitinib (a drug employed for treating gastrointestinal stromal tumors) was investigated using a novel theranostic system based on MIPs [77]. In this drug delivery system, rhodamine 6G was employed as a fluorescent marker for rapidly detecting sunitinib. A drug release of 67% was achieved in 6 h, which is significantly lower than the drug release of BTZ from the FMMIP-BTZ developed in this work. This significant difference in drug release capacities can be ascribed to the nature of the drugs and materials used for MIP construction.

Additionally, many systems show extended-release capacities greater than 6 h, as summarized in Table 2 [28, 78–80]. As discussed above, the newly synthesized FMMIP-BTZ nanocomposite developed in this work exhibited a remarkable release capacity of 80.5% for BTZ in 6 h at room temperature. It also showed a rapid response to an EMF and offered facile detection by fluorimeter. This study may help develop DDSs, which are advantageous in delivering drugs and/or hormones for humans and animals.

**Table 2:** Comparative study of MIP-based drug delivery systems employed for cancer therapy

No.	DDS type	Imprinting approach	Active drug	Drug release (%), time (h), T (°C)	Biocompatibility/ Biodegradability	Development Stage	Ref
1	Magnetic MIPs	NIPAM, CD, & Fe₃O₄	Curcumin	45, 7, 25	Yes/No	In vitro studies	[58]
2	MIPs beads @R6G	MAA/EGDMA/AIBN/ ACN/R6G/AA	Sunitinib	67, 6, 37	Yes/No	In vitro studies	[77]
3	MIP-coated Fe₃O₄ @ Si nanospheres	NIPA/MBA/APS/Water	5-Fluorouracil	70, 1.6, 37	Yes/No	In vitro Studies	[81]
4	MIP-coated Fe₃O₄	Non-covalent imprinting Dopamine/water	5-Fluorouracil	80, 4, 37	Yes/Yes	In vitro & in vivo studies	[82]
5	MIP-coated Fe₃O₄ @ SiO₂ @FITC/MPS	Non-covalent imprinting Tannic acid/AIBN/ Hexadecane + SDS in water	5-Fluorouracil	70, 80, 37	Yes/Yes	In vitro & in vivo studies	[28]
6	MIP-coated Fe₃O₄ NPs	Non-covalent imprinting NIPA/EDMA/AIBN/ Water + ethanol	Doxorubicin	70, 144, 37	Yes/No	In vitro studies	[78]
7	MIP-coated mesoporous silica NPs	Non-covalent imprinting MABHD, EDMA/DMAP AIBN/ethanol	Doxorubicin	72, 12, 37	Yes/No	In vitro release Studies	[80]
8	MIP-coated Fe₃O₄ NPs	Non-covalent imprinting Dopamine/water	Doxorubicin	90, 8, 37	Yes/Yes	In vivo studies	[83]
9	MIP-GO quantum dot microspheres	Non-covalent imprinting MAA/EDMA/AIBN/water	Doxorubicin	36.54, 3	No/No	In vitro studies	[79]
10	FMMIP-BTZ	Fe₃O₄ @SiO₂@FITC@PDA	Bortezomib	80.5, 6, 25	Yes/No	In vitro studies	Current study

*CD* _-cyclodextrin, *GO* Graphene oxide, *PDA* Polydopamine, *FITC* Fluorescein isothiocyanate, *MAA* Methacrylic acid, *EGDMA* Ethylene glycol dimethacrylate, *MABHD* 2-Methacrylester, *DMAP N*,N Dimethylaminopyridine, *AIBN* Azobisisobutyronitrile, hydroxyethyl disulfide, *NIPA N*-Isopropylacrylamide, *MPS* Methacryloxypropyl trimethoxysilane, *SDS* Sodium dodecyl sulfate, *ACN* Acetonitrile, *AA* Acrylic acid, *APS* Ammonium persulfate, *NIPAM N*-isopropylacrylamide,* R6G* rhodamine 6G

### Limitation

The current research shows the successful synthesis of a multifunctional FMMIP-BTZ nanocarrier, exhibiting release control, magnetic separation property, fluorescence tracking, and preliminary dermal compatibility, however, there are certain limitations. This current investigation does not contain cancer-cell uptake studies, cytotoxicity evaluation against tumour models, hemocompatibility evaluation, pharmacokinetic evaluation, biodistribution studies or in vivo evaluation of anti-tumour efficacy. Moreover, molecular docking and molecular dynamics simulation could give some mechanistic information, but it cannot replace experimental biological validation. Hence, biomedical potential of FMMIP-BTZ is still preliminary and needs to be explored by extensive and in-depth cell and animal studies.

## Conclusion

The surface imprinting and nanotechnology were combined to prepare a multifunctional fluorescent-magnetic molecularly imprinted polymer nanocomposite (Fe₃O₄ @SiO₂@FITC@PDA-BTZ) for selective recognition and controlled release of bortezomib (BTZ). SEM, TEM, XRD, FTIR, and TGA structural and physicochemical characterizations confirmed the successful formation of the nanocomposite. The developed FMMIP-BTZ showed good selectivity for BTZ recognition, magnetic separability under the tested conditions, and a high BTZ release capacity (80.5%). The release behavior was found to be zero-order kinetic which indicates the controlled and sustained release of BTZ for 24 h. Dual fluorescence was used for monitoring the BTZ and nanocomposite components and initial dermal irritation tests showed a good compatibility even under the tested conditions. Molecular docking and molecular dynamic simulation studies were also done and gave a computational information of the molecular interaction by BTZ. FMMIP-BTZ is a multifunctional nanoplatform with high potential for molecular recognition, fluorescence tracking, magnetic separation, and controlled drug release, yet it needs to be further investigated in biological settings to assess its potential therapeutic applications.

## Supplementary Information

Below is the link to the electronic supplementary material.

Supplementary Material 1

## Data Availability

The datasets used and analyzed during the current study are available from the corresponding author upon reasonable request.

## References

[1] Bray F, et al. Global cancer statistics 2022: GLOBOCAN estimates of incidence and mortality worldwide for 36 cancers in 185 countries. Cancer J Clin. 2024;74(3):229–63. 10.3322/caac.21834 38572751

[2] Sung H, et al. Global cancer statistics 2020: GLOBOCAN estimates of incidence and mortality worldwide for 36 cancers in 185 countries. Cancer J Clin. 2021;71(3):209–49. 10.3322/caac.21660 33538338

[3] Kim B, et al. Tuning payload delivery in tumour cylindroids using gold nanoparticles. Nat Nanotechnol. 2010;5(6):465–72. 10.1038/nnano.2010.58 PMC2881185 20383126

[4] Raza H, et al. Synthesis and characterization of Hyaluronic Acid (HA) modified polymeric composite for effective treatment of wound healing by transdermal drug delivery system (TDDS). Sci Rep. 2023;13(1):13425. 10.1038/s41598-023-40593-9 PMC10435553 37591923

[5] Fei Y, et al. Hierarchical integration of degradable mesoporous silica nanoreservoirs and supramolecular dendrimer complex as a general-purpose tumor-targeted biomimetic nanoplatform for gene/small-molecule anticancer drug co-delivery. Nanoscale. 2020;12(30):16102–12. 10.1039/d0nr03978k 32724952

[6] Sztandera K, Gorzkiewicz M, Klajnert-Maculewicz B. Nanocarriers in photodynamic therapy—in vitro and in vivo studies. Wiley Interdisciplinary Reviews: Nanomed Nanobiotechnol. 2020;12(3):e1509. 10.1002/wnan.1599 31692285

[7] Siriviriyanun A, et al. Preparation of graphene oxide/dendrimer hybrid carriers for delivery of doxorubicin. Chem Eng J. 2015;281:771–81.

[8] Chung YH, Cai H, Steinmetz NF. Viral nanoparticles for drug delivery, imaging, immunotherapy, and theranostic applications. Adv Drug Deliv Rev. 2020;156:214–35. 10.1016/j.addr.2020.06.024 PMC7320870 32603813

[9] Mei C, et al. Photothermal-controlled nanotubes with surface charge flipping ability for precise synergistic therapy of triple‐negative breast cancer. Adv Funct Mater. 2018;28(45):1805225.

[10] Ram Prasad RP et al. *Polymer-based nanoparticles for drug delivery systems and cancer therapeutics*, in *Natural polymers for drug delivery*. 2017, CABI Wallingford UK. pp. 53–70.

[11] Li P, et al. Synthetic glycopolypeptide micelle for targeted drug delivery to hepatic carcinoma. Polymers. 2018;10(6):611. 10.3390/polym10060611 PMC6403909 30966645

[12] Han Y, et al. Nanostructured lipid carriers as novel drug delivery system for lung cancer gene therapy. Pharm Dev Technol. 2016;21(3):277–81. 10.3109/10837450.2014.996900 25560648

[13] Tong X, et al. Recent advances in natural polymer-based drug delivery systems. Reactive Funct Polym. 2020;148:104501.

[14] Li L, Yang W-W, Xu D-G. Stimuli-responsive nanoscale drug delivery systems for cancer therapy. J Drug Target. 2019;27(4):423–33. 10.1080/1061186X.2018.1519029 30173577

[15] Guo Z, et al. pH-Responsive charge switchable PEGylated ε-poly-l-lysine polymeric nanoparticles-assisted combination therapy for improving breast cancer treatment. J Controlled Release. 2020;326:350–64. 10.1016/j.jconrel.2020.07.030 32707209

[16] Fu J, et al. Synthesis of amphiphilic AIE fluorescent nanoparticles via CO2 involved multicomponent reaction and its biological imaging potential. Dyes Pigm. 2023;210:110990.

[17] Cui Y, Ding L, Ding J. Recent advances of magnetic molecularly imprinted materials: From materials design to complex sample pretreatment. TRAC Trends Anal Chem, 2022: 147: p. 116514. 10.1016/j.trac.2021.116514

[18] Sun J-T, Hong C-Y, Pan C-Y. Recent advances in RAFT dispersion polymerization for preparation of block copolymer aggregates. Polym Chem. 2013;4(4):873–81.

[19] Piletska EV, et al. Influence of the polymerization conditions on the performance of molecularly imprinted polymers. Macromolecules. 2009;42(14):4921–8.

[20] Kupai J, et al. Long-term stability and reusability of molecularly imprinted polymers. Polym Chem. 2017;8(4):666–73. 10.1039/c6py01853j PMC5361172 28496524

[21] Marfà J, et al. Magnetic-molecularly imprinted polymers in electrochemical sensors and biosensors. Anal Bioanal Chem. 2021;413(24):6141–57. 10.1007/s00216-021-03461-x 34164705

[22] Hasanah AN, et al. Factors affecting preparation of molecularly imprinted polymer and methods on finding template-monomer interaction as the key of selective properties of the materials. Molecules. 2021;26(18):5612. 10.3390/molecules26185612 PMC8470890 34577083

[23] Loghmani MH, Shojaie AF, Hosseini SA. Glutathione-responsive hydrogel and molecularly imprinted polymer nanospheres: new aspect on cisplatin delivery. J Ind Eng Chem. 2021;96:98–108.

[24] Ali Z, et al. Biodegradable magnetic molecularly imprinted anticancer drug carrier for the targeted delivery of docetaxel. ACS omega. 2022;7(32):28516. 10.1021/acsomega.2c03299 PMC9386705 35990493

[25] Yew YP, et al. Green biosynthesis of superparamagnetic magnetite Fe3O4 nanoparticles and biomedical applications in targeted anticancer drug delivery system: A review. Arab J Chem. 2020;13(1):2287–308.

[26] Bermejo M, et al. Oral controlled release dosage forms: dissolution versus diffusion. Expert Opin Drug Deliv. 2020;17(6):791–803. 10.1080/17425247.2020.1750593 32248713

[27] Younis MA, et al. Clinical translation of nanomedicines: challenges, opportunities, and keys. Adv Drug Deliv Rev. 2022;181:114083. 10.1016/j.addr.2021.114083 34929251

[28] Asadi E, et al. In vitro/in vivo study of novel anti-cancer, biodegradable cross-linked tannic acid for fabrication of 5-fluorouracil-targeting drug delivery nano-device based on a molecular imprinted polymer. RSC Adv. 2016;6(43):37308–18.

[29] Wu H-B, Liu S-H, Cao C-R. Influence and assessment of AIBN on thermal hazard under process situations. J Therm Anal Calorim. 2021;144(4):1547–55.

[30] May J, Sayle R. *Encoding reactive chemical hazards and incompatibilities in an alerting system*. in *ABSTRACTS OF PAPERS OF THE AMERICAN CHEMICAL SOCIETY*. 2015. AMER CHEMICAL SOC 1155 16TH ST, NW, WASHINGTON, DC 20036 USA.

[31] Greim H, et al. Assessment of structurally related chemicals: toxicity and ecotoxicity of acrylic acid and acrylic acid alkyl esters (acrylates), methacrylic acid and methacrylic acid alkyl esters (methacrylates). Chemosphere. 1995;31(2):2637–59. 10.1016/0045-6535(95)00136-v 7663949

[32] Cutaia A, Alberti G. Green Synthesis of Molecularly Imprinted Polymers: Advances Toward Sustainable Materials. Polymers. 2026;18(4):512. 10.3390/polym18040512 PMC12944128 41754701

[33] Kazemi A, Panahi HA, Safaeijavan R. Thermosensitive molecularly imprinted poly (1-vinyl‐2‐pyrrolidone/methyl methacrylate/N‐vinylcaprolactam) for selective extraction of imatinib mesylate in human biological fluid. J Sep Sci. 2020;43(3):614–21. 10.1002/jssc.201900535 31691505

[34] Panahi HA, Alaei HS. β-Cyclodextrin/thermosensitive containing polymer brushes grafted onto magnetite nano-particles for extraction and determination of venlafaxine in biological and pharmaceutical samples. Int J Pharm. 2014;476(1–2):178–84. 10.1016/j.ijpharm.2014.09.051 25278039

[35] Baimani N, et al. Ultrasensitive separation of methylprednisolone acetate using a photoresponsive molecularly imprinted polymer incorporated polyester dendrimer based on magnetic nanoparticles. J Sep Sci. 2019;42(7):1468–76. 10.1002/jssc.201801093 30689289

[36] Yan J, et al. Polydopamine spheres as active templates for convenient synthesis of various nanostructures. Small. 2013;9(4):596–603. 10.1002/smll.201201064 23117928

[37] Lan F, et al. Polydopamine-based superparamagnetic molecularly imprinted polymer nanospheres for efficient protein recognition. Colloids Surf B. 2014;123:213–8. 10.1016/j.colsurfb.2014.09.018 25288533

[38] Chen J, et al. Fabrication of high-performance magnetic lysozyme-imprinted microsphere and its NIR-responsive controlled release property. ACS Appl Mater Interfaces. 2015;7(51):28606–15. 10.1021/acsami.5b10126 26642106

[39] Laurent S, et al. Magnetic iron oxide nanoparticles: synthesis, stabilization, vectorization, physicochemical characterizations, and biological applications. Chem Rev. 2008;108(6):2064–110. 10.1021/cr068445e 18543879

[40] Stöber W, Fink A, Bohn E. Controlled growth of monodisperse silica spheres in the micron size range. J Colloid Interface Sci. 1968;26(1):62–9.

[41] Huang J et al. Molecularly imprinted polymer coating with fluorescence on magnetic particle. Macromolecular Research.2013. 21(9): pp. 1021– 1028. 10.1007/s13233-013-1134-2.

[42] Ali Z, et al. Synthesis and characterization of a fluorescent magnetic molecularly imprinted polymer for enhanced adsorption and selective extraction of pesticides from agricultural waters. Front Nanatechnol. 2024;6:1516133.

[43] Hu M et al. Polydopamine-based molecularly imprinting polymers on magnetic nanoparticles for recognition and enrichment of ochratoxins prior to their determination by HPLC. *Microchimica Acta.* 2018. 185(6): 300. 10.1007/s00604-018-2826-2 29766346

[44] Khaneghah SS, Sohrabi N, Mohammadi R. Synthesis and characterization of magnetic molecularly imprinted polymer of polydopamine/graphene oxide as drug carrier for rivastigmine. J Mol Liq. 2023;391:123238.

[45] Lamaoui A, Lahcen AA, Amine A. Unlocking the potential of molecularly imprinted polydopamine in sensing applications. Polymers. 2023;15(18):3712. 10.3390/polym15183712 PMC10536926 37765566

[46] Trott O, Olson AJ. AutoDock Vina: improving the speed and accuracy of docking with a new scoring function, efficient optimization, and multithreading. J Comput Chem. 2010;31(2):455–61. 10.1002/jcc.21334 PMC3041641 19499576

[47] Csizmadia P. *MarvinSketch and MarvinView: molecule applets for the World Wide Web.* 1999.

[48] Spessard GO. ACD Labs/LogP dB 3.5 and ChemSketch 3.5. J Chem Inf Comput Sci. 1998;38(6):1250–3.

[49] Morris GM, et al. AutoDock4 and AutoDockTools4: Automated docking with selective receptor flexibility. J Comput Chem. 2009;30(16):2785–91. 10.1002/jcc.21256 PMC2760638 19399780

[50] Visualizer DS. Discovery Studio Visualizer, version 2.0. San Diego, CA: Accelrys Software Inc. 2005.

[51] Eastman P, et al. OpenMM 7: Rapid development of high performance algorithms for molecular dynamics. PLoS Comput Biol. 2017;13(7):e1005659. 10.1371/journal.pcbi.1005659 PMC5549999 28746339

[52] Kim S, et al. PubChem 2025 update. Nucleic Acids Res. 2025;53(D1):D1516–25. 10.1093/nar/gkae1059 PMC11701573 39558165

[53] Baby K, et al. Computational drug repurposing of Akt-1 allosteric inhibitors for non-small cell lung cancer. Sci Rep. 2023;13(1):7947. 10.1038/s41598-023-35122-7 PMC10188557 37193898

[54] Elfiky AA, et al. Simulation of gold nanoparticle movement through normal and cancer cell membranes. Comput Biol Med. 2023;164:107363. 10.1016/j.compbiomed.2023.107363 37595520

[55] Bojdi MK, et al. Application of magnetic lamotrigine-imprinted polymer nanoparticles as an electrochemical sensor for trace determination of lamotrigine in biological samples. *RSC Advances**.*2016. 6(38): pp. 32374–80.

[56] Ali Z, et al. Synthesis of green fluorescent cross-linked molecularly imprinted polymer bound with anti-cancerous drug (docetaxel) for targeted drug delivery. Polym Bull. 2024;81(1):679–96.

[57] Chen X, et al. Alkalinity triggered the degradation of polydopamine nanoparticles. Polym Bull. 2021;78(8):4439–52.

[58] Sedghi R, Yassari M, Heidari B. Thermo-responsive molecularly imprinted polymer containing magnetic nanoparticles: Synthesis, characterization and adsorption properties for curcumin. Colloids Surf B. 2018;162:154–62. 10.1016/j.colsurfb.2017.11.053 29190466

[59] Fizir M, et al. A mini review on molecularly imprinted polymer based halloysite nanotubes composites: innovative materials for analytical and environmental applications. Reviews Environ Sci Bio/Technology. 2020;19(2):241–58.

[60] Bodoki AE, Iacob B-C, Bodoki E. Perspectives of molecularly imprinted polymer-based drug delivery systems in cancer therapy. Polymers. 2019;11(12):2085. 10.3390/polym11122085 PMC6960886 31847103

[61] Hamid M, et al. Inhibitory potential of triazines and hydrazinyl thiazole substituted chromones against the HslVU protease/chaperone complex, a novel drug target. Eur Rev Med Pharmacol Sci. 2022;26(22):8567–75. 10.26355/eurrev_202211_30392 36459037

[62] Feng Y-x, et al. Separation, identification, and molecular docking of tyrosinase inhibitory peptides from the hydrolysates of defatted walnut (Juglans regia L.) meal. Food Chem. 2021;353:129471. 10.1016/j.foodchem.2021.129471 33730668

[63] Abdelsattar AS, Dawoud A, Helal MA. Interaction of nanoparticles with biological macromolecules: A review of molecular docking studies. Nanotoxicology. 2021;15(1):66–95. 10.1080/17435390.2020.1842537 33283572

[64] Rodrigues P, et al. COX 2-inhibitors; a thorough and updated survey into combinational therapies in cancers. Med Oncol. 2024;41(1):41. 10.1007/s12032-023-02256-7 38165473

[65] Wang D, et al. Prognostic significance of cyclooxygenase-2 protein in pancreatic cancer: a meta-analysis. Tumor Biology. 2014;35(10):10301–7. 10.1007/s13277-014-2260-y 25034525

[66] Wilgus TA, et al. Topical application of a selective cyclooxygenase inhibitor suppresses UVB mediated cutaneous inflammation. Prostaglandins Other Lipid mediat. 2000;62(4):367–84. 10.1016/s0090-6980(00)00089-7 11060900

[67] Knoops B, et al. Multiple roles of peroxiredoxins in inflammation. Mol Cells. 2016;39(1):60–4. 10.14348/molcells.2016.2341 PMC4749876 26813661

[68] El-Abaseri TB, et al. The epidermal growth factor receptor increases cytokine production and cutaneous inflammation in response to ultraviolet irradiation. Int Sch Res Notices. 2013;2013(1):848705. 10.1155/2013/848705 PMC3708437 23878744

[69] Melnikova VO, Ananthaswamy HN. Cellular and molecular events leading to the development of skin cancer. Mutat research/fundamental Mol Mech Mutagen. 2005;571(1–2):91–106. 10.1016/j.mrfmmm.2004.11.015 15748641

[70] Zomer HD, Trentin AG. Skin wound healing in humans and mice: Challenges in translational research. J Dermatol Sci. 2018;90(1):3–12. 10.1016/j.jdermsci.2017.12.009 29289417

[71] Roy T, et al. The PI3K-Akt-mTOR and associated signaling pathways as molecular drivers of immune-mediated inflammatory skin diseases: update on therapeutic strategy using natural and synthetic compounds. Cells. 2023;12(12):1671. 10.3390/cells12121671 PMC10297376 37371141

[72] Teng Y, et al. The PI3K/Akt pathway: emerging roles in skin homeostasis and a group of non-malignant skin disorders. Cells. 2021;10(5):1219. 10.3390/cells10051219 PMC8156939 34067630

[73] Ndunda EN. Molecularly imprinted polymers—A closer look at the control polymer used in determining the imprinting effect: A mini review. J Mol Recognit. 2020;33(11):e2855. 10.1002/jmr.2855 32529728

[74] Hayat M, et al. Targeted extraction of pesticide from agricultural run-off using novel molecularly imprinted polymeric pendants. J Ind Eng Chem. 2022;109:202–9.

[75] Hayat M, et al. Molecularly imprinted polymeric sorbent for targeted dispersive solid-phase microextraction of fipronil from milk samples. ACS omega. 2022;7(45):41437–48. 10.1021/acsomega.2c05217 PMC9670290 36406537

[76] Xiang X, et al. Multifiber solid-phase microextraction using different molecularly imprinted coatings for simultaneous selective extraction and sensitive determination of organophosphorus pesticides. J Sep Sci. 2020;43(4):756–65. 10.1002/jssc.201900994 31722132

[77] Parisi OI, et al. Molecularly imprinted polymers (MIPs) as theranostic systems for sunitinib controlled release and self-monitoring in cancer therapy. Pharmaceutics. 2020;12(1):41. 10.3390/pharmaceutics12010041 PMC7022407 31947815

[78] Kaamyabi S, Habibi D, Amini MM. Preparation and characterization of the pH and thermosensitive magnetic molecular imprinted nanoparticle polymer for the cancer drug delivery. Bioorg Med Chem Lett. 2016;26(9):2349–54. 10.1016/j.bmcl.2016.03.020 27020717

[79] Xu Y, et al. A novel controllable molecularly imprinted drug delivery system based on the photothermal effect of graphene oxide quantum dots. J Mater Sci. 2019;54(12):9124–39.

[80] Zhang K, et al. A pH/glutathione double responsive drug delivery system using molecular imprint technique for drug loading. Appl Surf Sci. 2016;389:1208–13.

[81] Li L, et al. Temperature and magnetism bi-responsive molecularly imprinted polymers: Preparation, adsorption mechanism and properties as drug delivery system for sustained release of 5-fluorouracil. Mater Sci engineering: C. 2016;61:158–68. 10.1016/j.msec.2015.12.027 26838836

[82] Hashemi-Moghaddam H, et al. Evaluation of magnetic nanoparticles coated by 5-fluorouracil imprinted polymer for controlled drug delivery in mouse breast cancer model. Int J Pharm. 2016;497(1–2):228–38. 10.1016/j.ijpharm.2015.11.040 26621688

[83] Zavareh S, et al. Synthesis of polydopamine as a new and biocompatible coating of magnetic nanoparticles for delivery of doxorubicin in mouse breast adenocarcinoma. Cancer Chemother Pharmacol. 2016;78:1073–84. 10.1007/s00280-016-3169-5 27752764

