# Supplementary material for: A magnetically recoverable, fluorescence-tracked, molecularly imprinted nanocarrier for controlled bortezomib release
Source: Discov Nano. 2026 Nov 12;21(1):629. doi: 10.1186/s11671-026-05039-x (PMC13760014; doi:10.1186/s11671-026-05039-x)
Supplement: Supplementary file 1 [file 11671_2026_5039_MOESM1_ESM.docx]

**A Magnetically Recoverable, fluorescence-Tracked, Molecularly Imprinted Nanocarrier for Controlled Bortezomib Release**

Taha Majid Mahmood Sheikh^1^, Zeeshan Ali^2*^, Hafiz Muhmmad Usman Abid^3^, Muhammad Hayat^4*^, Mehwish Raza^5^

^1^ School of Medical Sciences, Shandong Xiehe University, Jinan, 250109, P. R. China

^2^ Department of Chemistry NFC Institute of Engineering & Technology, Multan 60000, Pakistan

^3^Faculty of Pharmacy, Bahauddin Zakariya University, Multan 60800, Pakistan

^4^ Institute of Chemical Sciences, Bahauddin Zakariya University, Multan 60800, Pakistan

^5^ Multidisciplinary Research Lab, Bahria University Health Sciences Campus Karachi,

Karachi, Pakistan

Correspondence: [zeeshan.ali@mnsuam.edu.pk](mailto:zeeshan.ali@mnsuam.edu.pk) , [chaudary151@gmail.com](mailto:chaudary151@gmail.com)

**Table S1: Magnetization values of** Fe_3_O_4_, Fe_3_O_4_@SiO_2_@FITC NPs, and FMMIPs

| **Sr #** | **Materials** | **Magnetization emu/g** |
| --- | --- | --- |
| **1** | **Fe_3_O_4_** | **0.48** |
| **2** | **Fe_3_O_4_@SiO_2_@FITC NPs** | **0.17** |
| **3** | **FMMIPs** | **0.04** |

**Table S2:** Time-dependent release of BTZ from FMMIPs nanocomposite as a function of solution pH range from 4–7.4.

| **Sr No.** | **Time (h)** | **% Release at pH 7.4** | **% Release at pH 5.5** | **% Release at pH 4** |
| --- | --- | --- | --- | --- |
| 1 | 1 | 39.5 | 59.2 | 69.7 |
| 2 | 2 | 40.2 | 65.9 | 75.7 |
| 3 | 3 | 40.7 | 69.2 | 80.0 |
| 4 | 4 | 40.9 | 75.2 | 83.5 |
| 5 | 5 | 40.9 | 77.3 | 84.5 |
| 6 | 6 | 41.1 | 78.3 | 84.5 |

**Figure S1:**  **Average particle size of Fe_3_O_4_, Core shell, and MMIPs**

**Figure S2:** UV-vis absorption spectrum of different concentrations of FMMIPs

**Figure S3:** UV-vis absorption spectra for non-selective binding of BTZ by non-imprinted polymers.
